# Comparative Effectiveness and Safety of Low-Dose Oral Anticoagulants in Patients With Atrial Fibrillation

**DOI:** 10.3389/fphar.2021.812018

**Published:** 2022-01-14

**Authors:** Sylvie Perreault, Alice Dragomir, Robert Côté, Aurélie Lenglet, Simon de Denus, Marc Dorais, Brian White-Guay, James Brophy, Mireille E. Schnitzer, Marie-Pierre Dubé, Jean-Claude Tardif

**Affiliations:** ^1^ Faculty of Pharmacy, Université de Montréal, Montreal, QC, Canada; ^2^ Department of Urology, Faculty of Medicine, University McGill, Montreal, QC, Canada; ^3^ Faculty of Medicine, Department of Neurology and Neurosurgery, McGill University, Montreal, QC, Canada; ^4^ Laboratory MP3CV, Faculty of Pharmacy, University of Picardie Jules Verne, Amiens, France; ^5^ Pharmacy, Amiens University Medical Center, Amiens, France; ^6^ Montreal Heart Institute, Montreal, QC, Canada; ^7^ StatSciences Inc., Notre-Dame-de-l'Île-Perrot, QC, Canada; ^8^ Faculty of Medicine, Université de Montréal, Montreal, QC, Canada; ^9^ Faculty of Medicine, McGill University, Montreal, QC, Canada

**Keywords:** atrial fibrillation, oral anticoagulant, effectiveness outcomes, safety outcomes, low dose

## Abstract

**Aims:** Observational studies of various dose levels of direct oral anticoagulants (DOACs) for patients with atrial fibrillation (AF) found that a high proportion of patients received a dose lower than the target dose tested in randomized controlled trials. There is a need to compare low-dose DOACs with warfarin or other DOACs on effectiveness and safety.

**Methods:** Using administrative data from Quebec province, Canada, we built a cohort of new warfarin or DOAC users discharged from hospital between 2011 and 2017. We determined CHA_2_DS_2_-VASc and HAS-BLED scores, and comorbidities for 3-year prior cohort entry. The primary effectiveness endpoint was a composite of ischemic stroke/systemic embolism (SE), and secondary outcomes included a safety composite of major bleeding (MB) events and effectiveness composite (stroke/SE, death) at 1-year follow-up. We contrasted each low-dose DOAC with warfarin or other DOACs as references using inverse probability of treatment weighting to estimate marginal Cox hazard ratios (HRs).

**Results:** The cohort comprised 22,969 patients (mean age: 80–86). We did not find a significant risk reduction for the stroke/SE primary effectiveness endpoint for DOACs vs. warfarin; however, we observed a significantly lower risk for low-dose dabigatran vs. warfarin (HR [95%CI]: 0.59 [0.42–0.81]) for effectiveness composite, mainly due to a lower death rate. The differences in effectiveness and safety composites between low-dose rivaroxaban vs. warfarin were not significant. However, low-dose apixaban had a better safety composite (HR: 0.68 [0.53–0.88]) vs. warfarin. Comparisons of dabigatran vs. apixaban showed a lower risk of stroke/SE (HR: 0.53 [0.30–0.93]) and a 2-fold higher risk of MB. The MB risk was higher for rivaroxaban than for apixaban (HR: 1.58 [1.09–2.29]).

**Conclusions:** The results of this population-based study suggest that low-dose dabigatran has a better effective composite than warfarin. Compared with apixaban, low-dose dabigatran had a better effectiveness composite but a worse safety profile. Low-dose apixaban had a better safety composite than warfarin and other low-dose DOACs. Given that the comparative effectiveness and safety seem to vary from one DOAC to another, pharmacokinetic data for specific populations are now warranted.

## Introduction

Atrial fibrillation (AF) is known to cause embolic stroke, and the prevalence of AF is likely to increase (Colilla et al., 2013). Ischemic strokes associated with AF are more severe and more lethal than strokes in the absence of AF (McGrath et al., 2013). Oral anticoagulant (OAC) therapy with direct oral anticoagulants (DOACs, such as dabigatran, rivaroxaban, apixaban, and edoxaban) or vitamin K antagonists (e.g., warfarin) can effectively prevent ischemic events (including strokes) in patients with non-valvular AF (Hart et al., 2007; Culebras and Messé, 2014; January et al., 2014; Lip et al., 2018). The optimal use of warfarin becomes more difficult in older adults, since the latter have a greater risk of both thromboembolic and bleeding events (Samsa and Matchar, 2000; Fanikos et al., 2005; Miyasaka et al., 2006). The difficulties associated with warfarin use have led to the widespread acceptance of fast-acting DOACs, which target specific clotting factors. DOACs are associated with a lower risk of drug interactions, are less influenced by dietary factors, and constitute alternatives to warfarin for the prevention of stroke and systemic embolism (SE) in patients with non-valvular AF (January et al., 2014; Lip et al., 2018).

The use of DOACs in patients with non-valvular AF has been studied in large randomized controlled trials (RCTs) (Connolly et al., 2009; Granger et al., 2011; Patel et al., 2011; Xu et al., 2016). Compared with warfarin, DOACs were shown to be superior or comparable in terms of efficacy and had similar or lower bleeding rates—especially for intracranial hemorrhage (Connolly et al., 2009; Granger et al., 2011; Patel et al., 2011; Xu et al., 2016). Recent real-world, population-based studies of DOAC use by patients with AF (Maura et al., 2017; Perreault et al., 2020) found that a low dose was more prevalent than the standard dose used in RCTs (Perreault et al., 2020).

Extrapolating the RCT data on DOAC doses to clinical decision-making is limited by the small number of patients included in RCTs (Connolly et al., 2009; Connolly et al., 2010; Granger et al., 2011; Patel et al., 2011). Variability in treatment adherence and patient follow-up constitutes an additional challenge in clinical management and is not optimally reflected by the RCT results (Steinberg et al., 2013; Cutler et al., 2014). Hence, there is a need to compare various low-dose DOACs with warfarin and each other in terms of effectiveness and safety in patients with AF. To address this gap in our knowledge, we built a cohort of hospitalized patients with a primary or secondary diagnosis of AF and then compared low-dose DOACs with warfarin and with each other.

## Materials and Methods

### Data Source

We built a cohort using data in the Med-Echo administrative databases (hospital discharges), medical services, and public drug plans administered by the Régie de l’Assurance Maladie du Québec (RAMQ). The databases were linked using encrypted health insurance numbers. Information from these databases provides a complete picture of hospital admissions (Tamblyn et al., 1995; Tamblyn et al., 2000; Wilchesky et al., 2004; Eguale et al., 2010). The protocol was approved by an independent ethics committee at the University of Montreal.

### Population-Based Cohort

The cohort was designed using claims data from the Quebec RAMQ and Med-Echo databases. We identified adult patients with a primary or secondary diagnosis of AF (inpatient codes: ICD-9 427.3, 427.31, or 427.32 or ICD-10 I48) discharged alive from hospital into the community between January 1st, 2011, and December 31st, 2017 (Humphries et al., 2004; Perreault et al., 2018). For patients with multiple admissions with an AF diagnosis, only the first admission was analyzed. In previous validation studies, the diagnostic performance of ICD-9 codes for AF was relatively good, with median positive predictive values of over 80% (Jensen et al., 2012).

We next identified patients who had filled a new prescription of apixaban (2.5 mg twice daily), dabigatran (110 mg twice daily), rivaroxaban (15 mg once daily) or warfarin in the 12 months following hospital discharge. These new users had not been exposed to any OACs in the year before the index claim date. Eligible patients also had to have continuous health insurance coverage for at least 12 months before the index claim date. The date of the first OAC claim after hospital discharge was taken as the date of cohort entry.

We excluded patients with end-stage chronic kidney disease or a kidney transplant, patients on dialysis at any time in the 3 years before the index date, those having undergone hip or knee replacement surgery in the 6 weeks before the index date, and those with a diagnosis of deep vein thrombosis or pulmonary embolism at baseline. We also excluded patients with a coagulation deficiency or having undergone certain medical procedures (including cardiac catheterization, stent placement, a coronary artery bypass graft, medical procedures for cerebrovascular disease, or defibrillator implantation) in the 3 months prior to the index date. Lastly, we excluded patients having undergone a cardiac valvular replacement in the 5 years prior to cohort entry.

### Exposure to Oral Anticoagulants

We used fill dates and the number of days’ supply per prescription to establish the dates of the patients’ exposure to DOACs or warfarin. Patients were categorized as being on treatment if they had filled prescriptions within 30 days of the end of the previous treatment period. A gap of 30 days or less between treatments was allowed; this is a reasonable duration because of the DOACs’ short half-life *in vivo* (Perreault et al., 2020). Consequently, we chose 1 month as the allowable gap, which corresponds to an adherence of 92% or more over the fixed 12-month exposure assessment period.

### Outcomes

The primary effectiveness outcome was a primary diagnosis of ischemic stroke or systemic embolism (SE) after hospital admission for acute care during the 12-month follow-up period. The secondary outcomes were 1) a safety composite of major bleeding events (intracranial hemorrhage (ICH), gastrointestinal hemorrhage, and all other bleeding events), 2) a benefit/risk composite (stroke/SE, major bleeding, and all-cause mortality), 3) all-cause mortality, 4) an effectiveness composite (stroke/SE and all-cause mortality), and 5) major bleeding (intracranial hemorrhage and gastrointestinal bleeding only) over the same period of follow-up.

We identified outcomes using ICD-9 or ICD-10 codes for the primary diagnosis of inpatient claims (Supplementary Table S1). The positive predictive values were over 80% (Levy et al., 1999; Blais et al., 2012). These codes performed relatively well in previous validation studies (Tirschwell and Longstreth, 2002; Blais et al., 2012; Jensen et al., 2012; Thigpen et al., 2015). The definition of major bleeding has been published previously (Perreault et al., 2018).

### Demographic and Clinical Characteristics of the Study Population

We documented the demographic data at cohort entry. Social and economic deprivation was assessed using the Pampalon index (Pampalon et al., 2009). We determined the presence of comorbidities from specific ICD-9 or ICD-10 codes recorded during the hospital stay and those recorded for inpatient and outpatient diagnoses during the 3 years prior to the index date (Blais et al., 2012; Roy et al., 2020). Using the data on patient characteristics and associated comorbidities, we then assessed the CHA_2_DS_2_-VASc score (Supplementary Tables S2, S3), the modified HAS-BLED score (Supplementary Tables S2, S4) (Lip et al., 2010; Friberg et al., 2012; Pisters et al., 2010). and the Charlson Comorbidity Index(Deyo et al., 1992; D'Hoore et al., 1996). A frailty score (based on an appropriate risk assessment index for the elderly) was evaluated for the two years preceding cohort entry (Crane et al., 2010; Fillion et al., 2019). Lastly, we assessed the prescriptions filled for several medications in the 2 weeks preceding cohort entry. Although data on aspirin claims were recorded, possible over-the-counter purchases might have made this variable less reliable.

### Statistical Analyses

We used descriptive statistics to summarize the patients’ demographic and clinical characteristics as a function of the DOAC initially prescribed after discharge from hospital.

In order to balance the distribution of baseline patient characteristics between groups, an inverse probability of treatment weighting (IPTW) method was employed (Austin and Stuart, 2015; Allan et al., 2020). We created IPTW populations for the following contrasts: 1) low-dose dabigatran vs. warfarin; 2) low-dose rivaroxaban vs. warfarin; 3) low-dose apixaban vs. warfarin; 4) low-dose dabigatran vs. apixaban; 5) low-dose rivaroxaban vs. apixaban; 6) low-dose dabigatran vs. rivaroxaban. We used a multivariable logistic regression model to estimate the propensity score defined as the probability of being in the observed (actual) treatment group, conditional on all baseline covariates. The IPTW weights used the inverse of the propensity score. This weighting creates a pseudo-population in which there is balance across treatment groups with respect to covariates included in the model (Supplementary Table S5). The IPTW approach attempts to minimize the impact of confounding bias in observational studies by approximating a randomization process used in randomized clinical trials. All weights were stabilized by multiplying by the marginal probability of being in the treatment group.

Descriptive statistics were used to characterize the patients after weighting by IPTW. We estimated standardized differences in baseline characteristics between the treatment groups, where differences > 10% may suggest meaningful imbalance (Austin and Stuart, 2015). For descriptive analyses, we presented the pre- and post-weighted between-group comparisons. We reported the outcomes per 100 person-years for each treatment in each IPTW population.

Patients were followed from the index date until the earliest of the following events: outcome, being institutionalized or hospitalized for more than 15 days, discontinuation of treatment, or switching to another oral anticoagulant or to another dosage, end of study, or death, whichever came first. The censoring was handled by the Cox proportional hazards model. We contrasted each low-dose DOAC with both warfarin and each other low-dose as references using IPTW to estimate marginal Cox hazard ratios (HRs) for outcomes under treatment (UT). We constructed confidence intervals using the validated robust standard error.

### Sensitivity Analyses

We performed several sensitivity analyses of the effectiveness and safety composite outcomes for low-dose DOACs, relative to warfarin or to each other (Fralick et al., 2020). Firstly, we performed an intent-to-treat (ITT) analysis in which we removed the censoring criteria of drug discontinuation or switching, so that all patients were followed up for 365 days unless they were censored for another reason. Secondly, we calculated an E-value as a guide to the potential impact of unmeasured confounding (VanderWeele and Ding, 2017). The E-value indicates how strongly an unmeasured confounder should be associated with use of each low-dose DOAC (relative to warfarin or another DOAC) to change the observed effects on effectiveness or safety to null, depending on the measured covariates. Lastly, we assessed the risk of diabetes complications (primary code of hospitalization (ICD-9: 250.1-250.9, 357.2, 366.41; ICD-10: E10-E14 excluding E10.9, E11.9, E12.9, E13.0, E14.9) and pneumonia (ICD9 code: 480-488 ICD10: J09-J18) as negative control outcomes. And, we assessed the impact of temporal trends accounted in the analysis by including the date of cohort entry in the IPTW matching. All statistical analyses were performed using SAS software (version 9.4, SAS Institute Inc. Cary, NC, United States).

## Results

### Demographics and Clinical Characteristics of the Study Population

**FIGURE 1:**
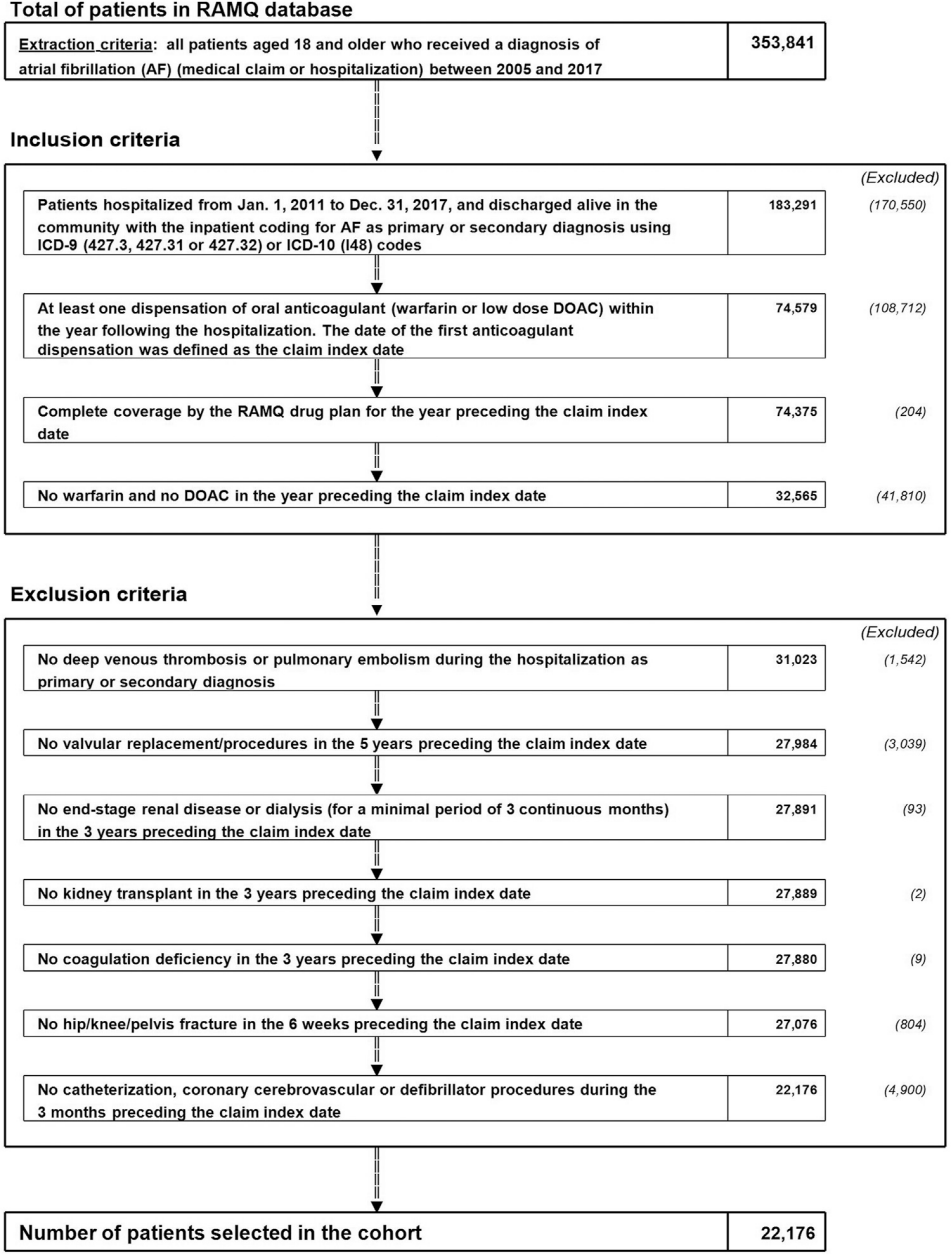
Study flow chart. AF: atrial fibrillation; OAC: oral anticoagulant; RAMQ: Régie d’Assurance Maladie du Québec (Quebec administrative databases).

A total of 22,176 patients with a confirmed diagnosis of AF received dabigatran (*n* = 1,929), rivaroxaban (*n* = 1,718), apixaban (*n* = 3,829) or warfarin (*n* = 14,700) (Figure 1). The characteristics of the study population for each DOAC after IPTW vs. warfarin are summarized in Table 1. In these groups, the mean age ranged from 80.2 to 82.2, and 55.8–58.9% were women. The characteristics of the study population for each DOAC after IPTW vs. the other DOACs are summarized in Table 2. In these groups, the mean age ranged from 82.0 to 85.3, and 59.6–66.1% were women. As shown in Supplementary Tables S5.1–S5.6, the absolute standardized differences in the IPTW populations were adequate.

**TABLE 1:** Demographic and clinical characteristics of OAC users from 2011 to 2018, after IPTW (DOACs vs. warfarin).

	IPTW dabigatran and warfarin populations		IPTW rivaroxaban and warfarin populations		IPTW apixaban and warfarin populations	
	Dabigatran 110 mg twice daily (*N* = 1,929)	Warfarin (*n* = 14,700)	Rivaroxaban 15 mg once daily (*N* = 1,718)	Warfarin (*n* = 14,700)	Apixaban 2.5 mg twice daily (*n* = 3,829)	Warfarin (*n* = 14,700)
Age, years, mean ± SD	80.2 (7.7)	80.2 (9.1)	80.7 ± 7.8	80.4 ± 9.1	82.2 ± 7.9	81.5 ± 9.1
Females (%)	56.8%	55.8%	57.0%	56.1%	58.9%	58.2%
Pampalon index: elevated social deprivation	26.7%	26.6%	26.5%	26.6%	26.6%	26.6%
Pampalon index: elevated material deprivation	25.7%	25.9%	25.6%	25.9%	25.7%	25.9%
CHA_2_DS_2_-VASc score (mean ± SD)*	4.0 ± 1.3	3.9 ± 1.4	4.0 ± 1.3	4.0 ± 1.4	4.2 ± 1.3	4.0 ± 1.4
CHA_2_DS_2_-VASc score 0–1	2.6%	3.9%	2.6%	3.7%	1.5%	3.2%
CHA_2_DS_2_-VASc score 2–3	32.8%	31.9%	30.7%	31.5%	28.2%	29.8%
CHA_2_DS_2_-VASc score 4	32.5%	31.1%	33.5%	31.3%	33.2%	31.9%
CHA_2_DS_2_-VASc score ≥5	32.1%	33.1%	33.2%	33.5%	37.1%	35.1%
HAS-BLED score (mean ± SD)*	3.3 ± 1.2	3.0 ± 1.3	3.4 ± 1.3	3.3 ± 1.3	3.4 ± 1.3	3.3 ± 1.3
HAS-BLED score <3	25.7%	27.4%	25.9%	26.8%	23.9%	26.6%
HAS-BLED score ≥3	74.3%	72.6%	74.1%	73.2%	76.1%	73.4%
Charlson comorbidity index*						
Charlson comorbidity index (mean ± SD)	4.9 ± 3.5	4.9 ± 3.4	5.2 ± 3.7	5.0 ± 3.4	5.3 ± 3.5	5.0 ± 3.4
Charlson comorbidity index (median [IQR])	4.0 (2.0–7.0)	4.0 (2.0–7.0)	5.0 (3.0–7.0)	4.0 (2.0–7.0)	5.0 (3.0–7.0)	4.0 (2.0–7.0)
Charlson comorbidity index < 4	40.9%	39.1%	36.6%	38.3%	34.0%	38.2%
Charlson comorbidity index ≥ 4	59.1%	60.9%	63.4%	61.7%	66.0%	61.8%
Frailty score (mean ± SD)	12.7 ± 6.9	12.6 ± 7.0	12.9 ± 6.9	12.6 ± 7.0	13.3 ± 6.9	12.9 ± 7.1
Robust (frailty score ≤ -1)	0%	0%	0%	0%	0%	0%
Well (frailty score: 0–3)	6.7%	8.0%	6.9%	7.9%	5.5%	7.2%
Well/comorbidities (frailty score: 4–8)	24.2%	25.4%	23.7%	25.2%	24.6%	24.6%
Pre-frail (frailty score: 9–15)	35.2%	33.0%	35.6%	33.1%	33.6%	33.5%
Frail (frailty score: ≥16)	33.9%	33.6%	33.8%	33.8%	36.3%	34.7%
Hypertension	84.4%	84.6%	86.0%	84.8%	86.1%	84.7%
Coronary artery disease	59.3%	59.1%	60.3%	59.4%	59.8%	58.8%
Acute myocardial infarction	14.0%	15.0%	16.5%	15.6%	17.1%	15.9%
Chronic heart failure	41.2%	43.1%	44.5%	43.6%	46.1%	44.0%
Cardiomyopathy	5.6%	6.2%	6.2%	6.3%	5.7%	6.1%
Other cardiac rhythm disorders	20.3%	20.7%	20.2%	20.2%	19.7%	20.1%
Valvular heart disease	22.3%	22.3%	22.0%	22.6%	23.2%	22.8%
Stroke/Transient ischemic attack	21.5%	21.4%	20.7%	20.9%	22.1%	20.8%
Peripheral vascular (arterial) disease	23.3%	24.4%	25.6%	24.7%	25.9%	24.4%
Dyslipidemia	51.8%	53.4%	53.2%	53.5%	53.2%	52.7%
Diabetes	35.0%	37.9%	38.8%	38.0%	37.7%	36.8%
Major bleeding	31.8%	32.4%	34.8%	32.8%	36.1%	33.1%
Major intracranial bleeding	3.6%	3.4%	4.4%	3.4%	5.1%	3.4%
Major gastrointestinal bleeding	8.9%	8.1%	8.4%	8.2%	9.3%	8.1%
Major bleeding at other sites	24.3%	25.4%	27.2%	25.9%	27.4%	26.4%
Chronic renal failure	42.6%	43.3%	49.0%	45.1%	51.3%	45.9%
Chronic renal failure ≤30 ml/min	5.7%	7.4%	7.2%	7.6%	8.6%	7.2%
Acute renal failure	25.1%	27.6%	30.9%	29.1%	33.6%	29.5%
Liver disease	2.2%	2.2%	2.6%	2.2%	2.4%	2.1%
Chronic obstructive pulmonary disease/asthma	37.6%	38.5%	40.5%	38.8%	37.6%	37.9%
*Helicobacter pylori* infection	0.8%	0.8%	1.5%	0.8%	0.6%	0.8%
Depression	11.8%	11.5%	11.0%	11.4%	11.3%	11.5%
Medical procedures*						
Cardiac catheterization	3.5%	3.8%	3.9%	3.8%	3.8%	3.7%
Percutaneous coronary intervention—stent	3.6%	2.9%	3.3%	3.0%	2.9%	2.8%
Coronary artery bypass grafting	1.0%	0.7%	0.9%	0.7%	0.5%	0.6%
Medical procedures for cerebrovascular disease	1.0%	1.1%	1.3%	1.1%	1.3%	1.0%
Medical procedures for a defibrillator	0.8%	0.5%	0.1%	0.4%	0.0%	0.4%
Medications (2 weeks prior cohort entry)						
Statin	46.8%	47.4%	47.5%	47.3%	46.0%	46.4%
Antiplatelet agents (excluding low-dose ASA)	6.2%	6.0%	6.3%	6.1%	6.2%	6.1%
Low-dose ASA	31.8%	31.5%	31.3%	31.4%	30.9%	30.8%
Proton pump inhibitors	46.1%	45.8%	46.2%	45.7%	47.0%	45.7%
NSAIDs	1.4%	1.4%	1.3%	1.3%	1.3%	1.3%
Digoxin	14.6%	13.5%	12.9%	13.3%	12.3%	12.8%
Amiodarone or propafenone	9.9%	10.1%	10.4%	10.1%	9.7%	10.1%
Antidepressants	9.0%	8.7%	8.5%	8.7%	8.5%	8.8%
B-blockers	60.8%	62.2%	62.2%	62.4%	61.2%	62.9%
Calcium channel blockers	39.1%	39.6%	39.7%	39.8%	40.8%	39.9%
Renin-angiotensin system inhibitors	38.9%	38.2%	38.3%	37.8%	37.3%	37.3%
Diuretics	42.3%	43.4%	45.6%	44.1%	46.1%	44.2%
Loop diuretics	35.2%	36.2%	38.6%	36.8%	39.2%	37.3%
Antidiabetics	20.8%	22.4%	23.4%	22.5%	21.9%	21.7%
PGP inhibitor use^‡^	61.0%	61.6%	61.9%	61.9%	62.1%	61.7%
Medical services (in the year prior to entry,%)						
Number of visits to a specialist (mean ± SD)	1.3 ± 2.2	1.2 ± 2.3	1.2 ± 2.0	1.2 ± 2.1	1.2 ± 2.2	1.2 ± 2.4
Number of family physician visits (mean ± SD)	1.3 ± 3.0	1.3 ± 3.0	1.3 ± 2.9	1.3 ± 3.0	1.3 ± 2.8	1.3 ± 3.0
Number of emergency room visits (mean ± SD)	3.3 ± 2.8	3.2 ± 2.8	3.3 ± 2.6	3.2 ± 2.8	3.3 ± 2.6	3.2 ± 2.8
Hospital services (in the 3 years prior to entry,%)						
≥2 all-cause hospital admissions	61.8%	58.3%	59.3%	58.2%	57.4%	58.0%
Number of all-cause hospital admissions (mean admission (±SD)	2.4 ± 1.7	2.4 ± 1.8	2.4 ± 2.0	2.4 ± 1.8	2.4 ± 1.9	2.4 ± 1.9
Hospital length of stay (mean ± SD)	11.1 ± 14.2	10.8 ± 12.0	11.1 ± 13.4	10.8 ± 12.0	11.2 ± 13.2	11.2 ± 13.2

*In the 3 years to the cohort entry; ^‡^P-glycoprotein. IPTW: inverse probability of treatment weighting; ^†^Antidepressants: SSRIs (citalopram, escitalopram, fluoxetine, paroxetine, sertraline)

**TABLE 2:** Demographic and clinical characteristics of OACs users from 2011 to 2018, after IPTW (comparisons of DOACs).

	IPTW dabigatran and apixaban populations		IPTW rivaroxaban and apixaban populations		IPTW dabigatran and rivaroxaban populations	
	Dabigatran 110 mg twice daily (*N* = 1,929)	Apixaban 2.5 mg twice daily (*n* = 3,829)	Rivaroxaban 15 mg once daily (*N* = 1,718)	Apixaban 2.5 mg twice daily (*n* = 3,829)	Dabigatran 110 mg twice daily (*N* = 1,929)	Rivaroxaban 15 mg once daily (*N* = 1,718)
Age—mean ± SD	84.2 ± 6.6	84.2 ± 7.8	85.3 ± 6.7	85.2 ± 7.0	81.9 ± 7.0	82.0 ± 7.5
Female (%)	64.5%	64.9%	65.8%	66.1%	59.6%	59.8%
Pampalon index elevated social deprivation	26.6%	26.6%	26.5%	26.6%	26.6%	26.5%
Pampalon index elevated material deprivation	25.7%	25.7%	25.6%	25.7%	25.7%	25.6%
CHA_2_DS_2_-VASc score (mean ± SD)*	4.1 ± 1.2	4.1 ± 1.3	4.2 ± 1.2	4.2 ± 1.2	3.9 ± 1.2	3.9 ± 1.3
CHA_2_DS_2_-VASc score 0–1	0.9%	1.4%	0.8%	0.9%	1.7%	2.7%
CHA_2_DS_2_-VASc score 2–3	28.5%	28.4%	25.8%	26.1%	34.5%	33.3%
CHA_2_DS_2_-VASc score 4	36.4%	35.1%	37.4%	36.1%	35.5%	34.8%
CHA_2_DS_2_-VASc score ≥5	34.2%	35.1%	36.0%	36.9%	28.3%	29.2%
HAS-BLED score (mean ± SD)*	3.2 ± 1.2	3.2 ± 1.3	3.2 ± 1.3	3.2 ± 1.3	3.0 ± 1.2	3.1 ± 1.3
HAS-BLED score <3	28.7%	31.1%	29.4%	28.9%	31.9%	34.0%
HAS-BLED score ≥3	71.3%	68.9%	70.6%	71.1%	68.1%	66.0%
Charlson score*						
Charlson comorbidity index (mean ± SD)	4.6 ± 3.4	4.6 ± 3.3	4.8 ± 3.4	4.7 ± 3.4	4.4 ± 3.4	4.4 ± 3.4
Charlson comorbidity index (median [IQR])	4.0 (2.0–6.0)	4.0 (2.0–6.0)	4.0 (2.0–6.0)	4.0 (2.0–6.0)	4.0 (2.0–6.0)	4.0 (2.0–6.0)
Charlson comorbidity index <4	45.0%	43.2%	41.7%	41.2%	47.8%	48.0%
Charlson comorbidity index ≥4	55.0%	56.8%	58.3%	58.8%	52.2%	52.0%
Frailty score (mean ± SD)	13.1 ± 6.8	13.0 ± 7.0	13.3 ± 6.8	13.2 ± 6.9	12.2 ± 6.8	12.2 ± 6.7
Robust (frailty score ≤ −1)	0%	0%	0%	0%	0%	0%
Well (frailty score: 0–3)	6.3%	6.3%	5.3%	5.5%	8.0%	8.1%
Well/comorbidities (frailty score: 4–8)	23.1%	25.0%	22.8%	24.1%	26.1%	26.3%
Pre-frail (frailty score: 9–15)	35.0%	34.9%	37.9%	35.8%	35.2%	36.1%
Frail (frailty score: ≥16)	35.6%	33.8%	34.0%	34.6%	30.7%	29.5%
Hypertension	83.4%	83.1%	83.7%	83.9%	83.2%	82.9%
Coronary artery disease	52.4%	52.7%	53.2%	53.4%	52.5%	52.5%
Acute myocardial infarction	13.9%	14.4%	15.8%	15.8%	11.9%	12.2%
Chronic heart failure	40.2%	40.4%	42.1%	41.7%	36.7%	36.8%
Cardiomyopathy	4.5%	4.9%	5.0%	5.2%	5.0%	4.9%
Other cardiac rhythm disorders	20.1%	19.9%	18.3%	18.5%	20.7%	20.5%
Valvular heart disease	20.6%	20.5%	21.1%	21.2%	18.2%	18.2%
Stroke/Transient ischemic attack	21.6%	20.5%	19.6%	19.2%	20.7%	20.4%
Peripheral vascular (arterial) disease	20.3%	20.2%	21.0%	21.2%	20.2%	20.5%
Dyslipidemia	47.9%	49.2%	49.3%	49.6%	50.0%	49.7%
Diabetes	28.7%	29.4%	30.0%	29.7%	30.5%	29.9%
Major bleeding	33.5%	32.3%	33.1%	33.1%	29.3%	29.0%
Major intracranial bleeding	3.3%	5.0%	4.0%	4.6%	3.2%	3.7%
Major gastrointestinal bleeding	9.1%	7.9%	7.3%	8.2%	8.7%	7.1%
Major bleeding at other sites	26.0%	24.1%	26.4%	25.2%	21.9%	22.3%
Chronic renal failure	39.3%	38.3%	43.3%	43.5%	32.7%	32.8%
Chronic renal failure ≤30 ml/min	3.4%	2.8%	3.7%	3.4%	2.1%	2.2%
Acute renal failure	23.5%	23.0%	27.0%	27.1%	18.6%	18.7%
Liver disease	1.8%	1.8%	1.5%	1.7%	2.0%	1.9%
Chronic obstructive pulmonary disease/asthma	35.1%	34.4%	35.0%	35.2%	36.3%	36.1%
*Helicobacter pylori* infection	0.8%	0.6%	1.2%	0.6%	0.7%	1.2%
Depression	12.6%	12.8%	12.3%	12.4%	13.1%	13.1%
Medical procedures*						
Cardiac catheterization	2.9%	2.7%	2.7%	2.6%	2.9%	2.8%
Percutaneous coronary intervention—stent	2.3%	2.3%	2.5%	2.4%	2.4%	2.6%
Coronary artery bypass grafting	0.3%	0.2%	0.3%	0.3%	0.6%	0.6%
Medical procedures for cerebrovascular disease	1.0%	0.7%	0.7%	0.7%	0.9%	0.9%
Medical procedures for a defibrillator	0.2%	0.0%	0.02%	0.00%	0.3%	0.3%
Medications (2 weeks prior to entry)						
Statin	41.0%	41.9%	40.8%	41.3%	42.8%	43.0%
Antiplatelets agents exclusing low-dose ASA)	6.0%	5.5%	5.7%	5.7%	5.1%	4.9%
Low-dose ASA	28.3%	26.9%	26.5%	26.4%	27.7%	27.4%
Proton pump inhibitors	43.7%	43.8%	43.7%	43.6%	43.1%	42.9%
NSAIDs	1.3%	1.3%	1.1%	1.1%	1.3%	1.3%
Digoxin	11.9%	11.6%	11.1%	10.9%	12.9%	12.9%
Amiodarone or propafenone	10.4%	9.7%	9.6%	9.8%	10.1%	9.9%
Antidepressants	10.5%	10.1%	9.6%	9.8%	9.5%	9.2%
B-blockers	63.3%	63.4%	65.1%	64.7%	62.9%	63.1%
Calcium channel blockers	39.6%	38.8%	38.9%	39.2%	37.8%	37.5%
Renin-angiotensin system inhibitors	38.4%	37.0%	35.3%	35.6%	39.2%	39.3%
Diuretics	41.0%	40.7%	42.0%	42.4%	38.8%	38.7%
Loop diuretics	34.4%	34.1%	35.1%	35.5%	30.8%	30.8%
Antidiabetics	16.7%	17.2%	17.2%	17.0%	17.7%	17.1%
PGP inhibitor use^‡^	59.4%	59.8%	60.5%	60.4%	59.5%	59.3%
Medical services*						
Number of visits to a specialist (mean ± SD)	1.4 ± 2.9	1.3 ± 2.8	1.3 ± 2.5	1.3 ± 2.6	1.3 ± 2.6	1.3 ± 2.5
Number of family physician visits (mean ± SD)	1.3 ± 3.0	1.3 ± 3.0	1.3 ± 2.9	1.3 ± 3.0	1.4 ± 3.1	1.4 ± 3.0
Number of emergency room visits (mean ± SD)	3.2 ± 2.6	3.3 ± 2.7	3.3 ± 2.4	3.3 ± 2.6	3.2 ± 2.8	3.2 ± 2.5
Hospital services (in the year before entry,%)						
≥2 all-cause hospital admissions	59.2%	54.8%	56.5%	54.1%	58.3%	57.4%
Number of all-cause hospital admissions (mean admission (±SD)	2.3 ± 1.5	2.3 ± 1.7	2.2 ± 1.6	2.2 ± 1.7	2.3 ± 1.6	2.3 ± 1.7
Hospital length of stay (mean ± SD)	10.1 ± 10.9	10.0 ± 11.3	10.3 ± 11.5	10.3 ± 11.5	9.4 ± 11.0	9.3 ± 10.9

*In the 3 years to the cohort entry; ^‡^P-glycoprotein. IPTW: inverse probability of treatment weighting; ^†^Antidepressants: SSRIs (citalopram, escitalopram, fluoxetine, paroxetine, sertraline)

### Cumulative Incidence Rates

The annualized rates [95% confidence interval (CI)] for effectiveness and safety outcomes when comparing low-dose DOAC vs. warfarin in as-treated and intent-to-treat analyses after IPTW are shown in Supplementary Tables S6.1, S6.2. Similarly, the rates for low-dose DOACs vs. the other DOACs are shown in Supplementary Tables S6.3, S6.4.

### Effectiveness and Safety Outcomes of Direct Oral Anticoagulants vs. Warfarin

**FIGURE 2:**
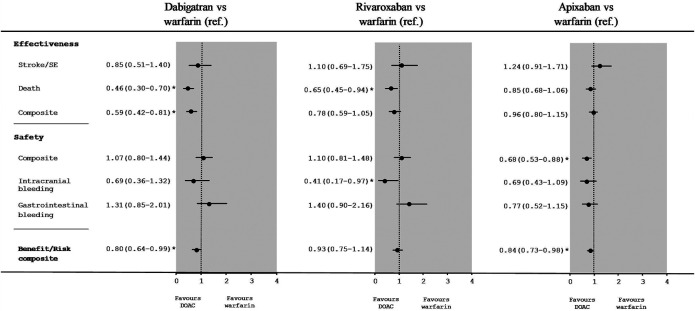
Hazard ratios [95%CI] for low-dose DOACs vs. warfarin in an as-treated analysis of effectiveness and safety outcomes after IPTW.

Figure 2 shows the HRs [95%CI] for the primary and secondary outcomes in IPTW populations taking low-dose DOACs vs. warfarin. The difference between dabigatran and warfarin was not statistically significant for the primary effectiveness outcome (stroke/SE) (HR [95%CI]: 0.85 [0.51–1.40]) and the safety composite (1.07 [0.80–1.44]). The HR [95%CI] for all-cause mortality was 0.45 (0.30–0.70]), the HR for the effectiveness composite was 0.59 [0.42–0.81] and the HR benefit/risk composite was 0.80 [0.64–0.99]). Similarly, the difference between rivaroxaban and warfarin was not statistically significant for the primary outcome (1.10 [0.69–1.75]), the safety composite (1.10 [0.81–1.48]) or the benefit/risk composite (0.93 [0.75–1.14]). The HR [95%CI] for all-cause mortality was 0.65 [0.45–0.94]). Lastly, there were no significant differences between apixaban and warfarin with regard to the primary outcome (HR [95%CI]: 1.24 [0.91–1.71]) but was significant for the safety composite (0.68 [0.53–0.88]) or the benefit/risk composite (0.84 [0.73–0.98]). The HR [95%CI] for all-cause mortality (0.85 [0.68–1.06]) was not statistically significant.

### Effectiveness and Safety Outcomes When Comparing Direct Oral Anticoagulants With Each Other

**FIGURE 3:**
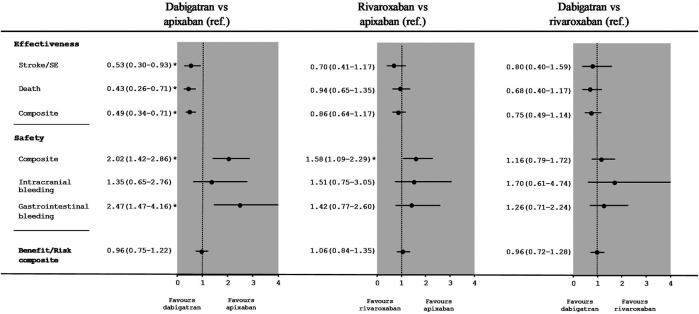
Hazard ratios [95%CI] for comparisons between low-dose DOACs in an as-treated analysis of effectiveness and safety outcomes after IPTW.

Figure 3 shows the HRs [95%CI] for the effectiveness and safety outcomes in IPTW populations taking one low-dose DOAC vs. another low-dose DOAC. There was a significant difference between low-dose dabigatran and low-dose apixaban with regard to stroke/SE (HR [95%CI]: 0.53 [0.30–0.93]) and the safety composite (2.02 [1.42–2.86]) but not the benefit/risk composite (0.96 [0.75–1.22]). The HR was 0.43 ([0.26–0.71) for all-cause mortality and 0.49 ([0.34–0.71]) for the effectiveness composite. The HR for gastrointestinal bleeding was 2.47 ([1.47–4.16]).

There were no significant differences between of low-dose rivaroxaban and low-dose apixaban with regard to stroke/SE (HR: 0.70; [0.41–1.17]) or the benefit/risk composite (1.06 ([0.84–1.35]) but rivaroxaban presented a worse safety profile (1.58 (1.09–2.29]). When comparing low-dose dabigatran with low-dose rivaroxaban, we did not find significant differences for stroke/SE (HR: 0.80; [0.40–1.59]), the safety composite (HR: 1.16; [0.79–1.72]), or the benefit/risk composite (HR: 0.96; [0.72–1.28]).

### Sensitivity Analyses

#### The Intent-To-Treat Analysis

Intention-to-treat (ITT) analyses of the IPTW populations followed up for 365 days gave consistent results (Supplementary Tables S7.1, S7.2) for all comparisons vs. warfarin or other DOACs.

#### The Impact of Unmeasured Confounders

For dabigatran vs. warfarin, the E-value corresponding to the CI boundary closest to 1 for the risk of death was 2.21 (Table 3). The observed HR for death might have been due to an unmeasured confounder that occurred 2.21 times more often in the dabigatran group than in the warfarin group and thus increased the death rate by a factor of 2. This assumes no correlation between the unmeasured confounder and the measured confounders used in the propensity score.

**TABLE 3:** E-values for significant comparisons (as-treated analysis) of low-dose DOACs with warfarin and with each other.

	Hazard ratio (95%CI)	E-value corresponding to the CI boundary closest to 1	E-value corresponding to the HR point estimate*
Low-dose dabigatran vs. warfarin			
Death	0.46* (0.30–0.70)	2.21	3.77
Effectiveness composite	0.55* (0.42–0.81)	1.77	3.04
Low-dose rivaroxaban vs. warfarin			
Death	0.65* (0.45–0.94)	2.45	2.45
Low-dose apixaban vs. warfarin			
Safety composite	0.68* (0.53–0.88)	1.53	2.30
Low-dose dabigatran vs. low-dose apixaban			
Stroke/systemic embolism	0.53* (0.30–0.93)	1.36	3.18
Death	0.43* (0.26–0.71)	2.17	4.08
Effectiveness composite	0.49* (0.34–0.71)	2.17	3.50
Gastrointestinal bleeding	2.47* (1.47–4.16)	2.30	4.38
Extracranial bleeding	2.30* (1.54–3.44)	2.45	4.03
Safety composite	2.02* (1.42–2.86)	2.19	3.46
Low-dose rivaroxaban vs. low-dose apixaban			
Extracranial bleeding	1.61* (1.04–2.49)	1.24	2.60
Safety composite	1.58* (1.09–2.29)	1.40	2.54

The E-value corresponding to the CI boundary closest to 1 for the various comparisons ranged from 1.36 to 3.62. Lastly, the E-value corresponding to the HR point estimates for the various comparisons ranged from 2.28 to 3.77—indicating that these situations are less likely to occur.

#### The Negative Control and Impact of the Temporal Trends

With regard to the rate per 100 person-years of pneumonia vs. warfarin and DOAC (Supplementary Table S8), none of the comparisons gave a significant HR. Moreover, the rates per 100 person-years of hospitalization for diabetes complications were quite similar for warfarin and DOACs, with no significant HRs. As expected, the results were similar in all the groups.

Similar results were observed for the overall comparative effectiveness and safety of each low-dose of DOACs versus warfarin (Supplementary Table S7.3) and also each low-dose of DOACs versus each other (Supplementary Table S7.4) with the inclusion of the base date of cohort entry in the IPTW matching. Some outcomes were marginally modified for the comparison versus warfarin mainly for rivaroxaban safety composite.

## Discussion

### Low-Dose Direct Oral Anticoagulants Compared With Warfarin

In our population-based study, we did not observe a significant reduction in the risk of the primary outcome (stroke/SE) for any of the low-dose DOACs (dabigatran, rivaroxaban, and apixaban) vs. warfarin. Moreover, there were no significant relationships with the safety profile, except for low-dose apixaban vs. warfarin (a 32% risk reduction for apixaban). With regard to the secondary outcomes, low-dose dabigatran and low-dose rivaroxaban were associated with a reduction (vs. warfarin) in the risk of all-cause mortality that ranged from 35 to 54%.

Our effectiveness and safety results for patients using low-dose dabigatran or warfarin are quite similar to those published for the RE-LY study (Connolly et al., 2009). Although a number of observational studies have compared dabigatran, rivaroxaban, and apixaban with warfarin in terms of effectiveness and safety, (Graham et al., 2016; Larsen et al., 2016; Hernandez et al., 2017; Nielsen et al., 2017; Li et al., 2018; Lopes et al., 2018; Graham et al., 2019) but few reported on the impact of low dose levels. However, Li et al. evaluated the effectiveness and safety of different dose levels of apixaban (vs. warfarin) with a similar study design and in a similar patient population. Apixaban 2.5 mg twice daily was associated with a lower risk of major bleeding (HR [95%CI]: 0.59 [0.49–0.71]) (Li et al., 2018). Our results are also consistent with those of another similar study in which (relative to warfarin) low-dose apixaban and low-dose dabigatran had no significant effects on a stroke/SE outcome, low-dose dabigatran was associated with a reduction in the risk of death, and low-dose apixaban presented a better safety profile for bleeding events (Rahme et al., 2021).

### Low-Dose Direct Oral Anticoagulants Compared With Each Other

Low-dose dabigatran presented a 47% difference in stroke/SE when compared with apixaban; however, it also had a less favorable safety profile, with more than a two-fold relative increase in the major bleeding risk. For low-dose rivaroxaban vs apixaban, we did not observe a significant difference in stroke/SE, although low-dose rivaroxaban had a less favorable safety composite. We noted no significant difference in the comparison of dabigatran and rivaroxaban for the effectiveness and safety outcomes.

The published RCTs did not perform head-to-head comparisons of different dose levels of DOACs. Furthermore, the observational studies of effectiveness and safety compared full dose levels of dabigatran, rivaroxaban, and apixaban—the three most widely used DOACs (Graham et al., 2019; Bonde et al., 2020; Fralick et al., 2020) A recent meta-analysis reported indirect comparisons, although the data on low-dose DOACs were scarce (Li et al., 2019). There were no significant differences in the stroke/SE outcome for rivaroxaban or dabigatran, when compared with apixaban. However, the risk of major bleeding was significantly higher for rivaroxaban than for apixaban (HR [95%CI]: 1.71 [1.51–1.94]). Moreover, a recent study reported nonsignificant differences in the stroke/SE outcome between low doses of dabigatran, rivaroxaban, and apixaban; however, apixaban had a better safety profile (Durand et al., 2021).

A recent placebo-controlled RCT in older Japanese patients with non-valvular AF (where a standard dose is not appropriate) found that edoxaban was efficacious in preventing stroke/SE and did not have any impact on major bleeding (other than gastrointestinal bleeding) (Okumura et al., 2020). In view of the lack of RCT data and the high prevalence of low-dose DOAC use, further studies of the effectiveness and safety of low-dose DOACs are clearly warranted. Moreover, given that the net benefit seems to vary from one DOAC to another, pharmacokinetic data for specific populations (such as those with higher risks of thrombosis and bleeding) must be generated by comparing plasma drug levels and factor Xa inhibition as a function of the dose level and the outcomes (Testa et al., 2018; Sukumar et al., 2019).

Our study had a number of strengths, including the large sample size and the analyses of the relative effectiveness and safety of low-dosage DOACs vs. warfarin and other DOACs in patients with AF. We assessed several clinical outcomes, in order to balance the overall benefits and risks. We used an IPTW population score model to build cohorts that were well balanced at baseline with regard to relevant factors, and we also performed several sensitivity analyses.

Our study also had some limitations. Firstly, this observational study was based on administrative data and so might have been subject to confounding bias by unadjusted factors (blood pressure control, laboratory values, international normalized ratio control, body weight, and estimated glomerular filtration rate) or to residual channeling bias. Secondly, most of our patients were older and ethnically white, and so our present results might not be generalizable to other patient settings (e.g., non-hospitalized individuals with AF), other age groups, or other ethnic groups (Shen et al., 2007). Lastly, residual bias is still possible—especially with regard to unmeasured variables and the healthy population effect.

The results of this population-based study suggest that low-dose dabigatran has a better effective composite than warfarin. Compared with apixaban, low-dose dabigatran had a better effectiveness composite but a worse safety profile. Low-dose apixaban had a better safety composite than warfarin and other low-dose DOACs. Studies of plasma drug levels and factor Xa inhibition as a function of the dose level and outcomes are now warranted, since the net benefit appears to vary from one DOAC to another.

## Data Availability

The original contributions presented in the study are included in the article/Supplementary Material, further inquiries can be directed to the corresponding author.
