# Supplementary material for: Comparative Effectiveness and Safety of Low-Dose Oral Anticoagulants in Patients With Atrial Fibrillation
Source: Front Pharmacol. 2022 Jan 14;12:812018. doi: 10.3389/fphar.2021.812018 (PMC8795908; doi:10.3389/fphar.2021.812018)
Supplement: Supplementary file 1 [file Table1.docx]

**Supplementary Tables:**

**Supplemental Table S1.** Definition of outcomes, according to the ICD-9 and ICD-10 codes recorded in the Med-Echo databases.

|  | ICD-9 codes | ICD-10 codes |
| --- | --- | --- |
| **Thromboembolic events** |  |  |
| **-Stroke** |  |  |
| Ischemic stroke (IS) | 433.xx, 434.xx, 436.0, 436.9 (primary diagnosis only using Med-Echo) | I63 except I63.6, I64 (primary diagnosis only using Med-Echo) |
| **- Systemic embolism (SE)** | 444.x, 557.0, 362.31, 362.32, 598.31 (primary diagnosis only using Med-Echo) | I74 (primary diagnosis only) |
| *Arterial embolism and thrombosis* | 444.x (primary diagnosis only using Med-Echo) | I74.0, I74.1, I74.2, I74.3, I74.5, I74.8, I74.9 (primary diagnosis only using Med-Echo) |
| *Ischemic colitis or mesenteric thromboembolism* | 557.0 (primary diagnosis only using Med-Echo) | K55.0 (primary diagnosis only using Med-Echo) |
| *Retinal artery thromboembolism* | 362.31, 362.32 (primary diagnosis only using Med-Echo) | H34.1, H34.2 (primary diagnosis only using Med-Echo) |
| *Renal artery thromboembolism* | 593.81 (primary diagnosis only using Med-Echo) | N28.0 (primary diagnosis only using Med-Echo) |
|  |  |  |
| **All-cause deaths** |  |  |
| **Myocardial infarction** | 410 | I21 |
| **Major bleedings** |  |  |
| **-Intracranial major bleeding** |  |  |
| Intracranial bleeding | 430, 431, 432.x, 852.x, 853.x (primary diagnosis or the first secondary diagnosis using Med-Echo) | I60, I61, I62, S06.3, S06.4, S06.5, S06.6 (primary diagnosis or the first secondary diagnosis using Med-Echo) |
| **-Major GI bleeding** |  |  |
| Upper gastrointestinal bleeding  (only using Med-Echo) | 456.1, 530.7, 531.0x, 531.2x, 531.4x, 531.6x, 532.0x, 532.2x, 532.4x. 532.6x, 533.0x. 533.2x, 533.4x, 533.6x, 534.0x, 534.2x, 534.4x, 534.6x, 535.1, 578.0 (primary diagnosis only using Med-Echo) | I85.0, K22.6, K25.0, K25.2, K25.4, K25.6, K26.0, K26.2, K26.4, K26.6, K27.0, K27.2, K27.4, K27.6, K28.0, K28.2, K28.4, K28.6, K29.0, K92.0 (primary diagnosis only using Med-Echo) |
| Upper gastrointestinal bleeding  (only using RAMQ) | 456.1, 530.7, 531.0x, 531.2x, 531.4x, 531.6x, 532.0x, 532.2x, 532.4x. 532.6x, 533.0x, 533.2x, 533.4x, 533.6x, 534.0x, 534.2x, 534.4x, 534.6x, 535.1, 578.0 RAMQ ICD-9 at an emergency room, and an endoscopic control of gastric or duodenal bleeding or upper gastrointestinal endoscopy including the esophagus, stomach, and either the duodenum and/or jejunum as appropriate with control of bleeding, any method (code 00691) within 7 days | I85.0, K22.6, K25.0, K25.2, K25.4, K25.6, K26.0, K26.2, K26.4, K26.6, K27.0, K27.2, K27.4, K27.6, K28.0, K28.2, K28.4, K28.6, K29.0, K92.0 RAMQ ICD-9 at an emergency room, and an endoscopic control of gastric or duodenal bleeding or upper gastrointestinal endoscopy including the esophagus, stomach, and either the duodenum and/or jejunum as appropriate with control of bleeding, any method (00691) within 7 days |
| Lower gastrointestinal bleeding | 562.02, 562.03, 562.12, 562.13, 569.3x, 569.85, 578.1x, 578.9 (primary diagnosis only using Med-Echo) | K57.11, K57.13, K57.31, K57.33, K62.5, K55.21, K92.1, K92.2 (primary diagnosis only using Med-Echo) |
| **-Other critical sites of major bleeding** |  |  |
| Gross hematuria | 599.7 (primary diagnosis only using Med-Echo) | R31 (primary diagnosis only using Med-Echo) |
| Hemoptysis | 786.3x (primary diagnosis only using Med-Echo) | R04.2, R04.89, R04.9 (primary diagnosis only using Med-Echo) |
| Vitreous hemorrhage | 379.23 (primary diagnosis only using Med-Echo) | H43.13 (primary diagnosis only using Med-Echo) |
| Urogenital bleeding | 626.2x and 280.0 (primary diagnosis only), 285.1 (primary diagnosis or the first secondary diagnosis) or 285.9 (primary diagnosis or the first secondary diagnosis using Med-Echo) | N92.0 and D50.0 (primary diagnosis only), D62 (primary diagnosis or the first secondary diagnosis), D64.9 (primary diagnosis or the first secondary diagnosis using Med-Echo) |
| Hemarthrosis | 719.1x (primary diagnosis only using Med-Echo) | M25.0x (primary diagnosis only using Med-Echo) |
| Hemopericardium | 423.0 (primary diagnosis only using Med-Echo) | I31.2 (primary diagnosis only using Med-Echo) |
| Hemoperitoneum | 568.8 (primary diagnosis only using Med-Echo) | K66.1 (primary diagnosis only using Med-Echo) |
| Hemorrhage not specified | 459.0x (primary diagnosis only using Med-Echo) | R58.0 (primary diagnosis only using Med-Echo) |
| Acute posthemorrhagic anemia | 285.1x (primary diagnosis only using Med-Echo) | D62 (primary diagnosis only using Med-Echo) |
