# Supplementary material for: Comparative Effectiveness and Safety of Low-Dose Oral Anticoagulants in Patients With Atrial Fibrillation
Source: Front Pharmacol. 2022 Jan 14;12:812018. doi: 10.3389/fphar.2021.812018 (PMC8795908; doi:10.3389/fphar.2021.812018)
Supplement: Supplementary file 2 [file Table8.docx]

**Supplementary Tables:**

**Supplemental Table S8.** Sensitivity analyses of low-dose DOACs for treated patients vs. nontreated (control) patients, after IPTW.

|  | Incident rate for DOAC* | Incident rate for  warfarin | HR vs. warfarin^†^ | P-Value | Incident rate for DOAC* | Incident rate for apixaban | HR vs. apixaban | P-  Value | Incident rate for DOAC* | Incident rate for rivaroxaban | HR vs. rivaroxaban | P-value |
| --- | --- | --- | --- | --- | --- | --- | --- | --- | --- | --- | --- | --- |
| **Pneumonia** |  |  |  |  |  |  |  |  |  |  |  |  |
| Dabigatran | 9.8 | 10.5 | 0.93; 0.77-1.12 | 0.4601 | 11.0 | 11.8 | 0.91; 0.74-1.12 | 0.3701 | 10.1 | 8.9 | 1.12; 0.86-1.45 | 0.3942 |
| Rivaroxaban | 9.9 | 10.7 | 0.94; 0.77-1.13 | 0.4956 | 10.5 | 12.3 | 0.85; 0.69-1.05 | 0.1286 | - | - | - | - |
| Apixaban | 12.1 | 11.0 | 1.12; 0.98-1.27 | 0.0882 | - | - | - | - | - | - | - | - |
| **Hospitalization for diabetes complications** | | | | | |  |  |  |  |  |  |  |
| Dabigatran | 0.41 | 0.70 | 0.58; 0.24-1.41 | 0.2275 | 0.42 | 0.50 | 0.82; 0.30-2.24 | 0.6950 | 0.39 | 0.33 | 1.20; 0.32-4.45 | 0.7876 |
| Rivaroxaban | 0.50 | 0.72 | 0.70; 0.30-1.62 | 0.4072 | 0.46 | 0.49 | 0.94; 0.34-2.56 | 0.8997 | - | - | - | - |
| Apixaban | 0.71 | 0.68 | 1.06; 0.63-1.77 | 0.8271 | - | - | - | - | - | - | - | - |

* Incident rate per 100-person-years: ^†^ Hazard ratio (HR) with the 95%CI.
