# Supplementary material for: Comparative Effectiveness and Safety of Low-Dose Oral Anticoagulants in Patients With Atrial Fibrillation
Source: Front Pharmacol. 2022 Jan 14;12:812018. doi: 10.3389/fphar.2021.812018 (PMC8795908; doi:10.3389/fphar.2021.812018)
Supplement: Supplementary file 3 [file Table7.docx]

**Supplementary Tables:**

**Supplemental Table S7.1.** Sensitivity analyses of comparative effectiveness and safety of low-dose DOACs versus warfarin in an as-treated analysis and an intent-to-treat analysis, after IPTW.

|  | **Analysis** | **Hazard Ratio (95%CI)** | **Hazard Ratio (95%CI)** | **Hazard Ratio (95%CI)** |
| --- | --- | --- | --- | --- |
|  |  | **Dabigatran vs. warfarin** | **Rivaroxaban vs. warfarin** | **Apixaban vs. warfarin** |
| ***Effectiveness*** |  |  |  |  |
| Stroke (ischemic only)/SE | UT | 0.85 (0.51-1.40) | 1.10 (0.69-1.75) | 1.24 (0.91-1.71) |
|  | ITT | 0.77 (0.48-1.22) | 1.22 (0.81-1.83) | 1.15 (0.86-1.55) |
| All-cause mortality | UT | 0.46 (0.30-0.70) | 0.65 (0.45-0.94) | 0.85 (0.68-1.06) |
|  | ITT | 0.79 (0.66-0.94) | 1.01 (0.86-1.20) | 1.10 (0.98-1.23) |
| Effectiveness composite | UT | 0.59 (0.42-0.81) | 0.78 (0.59-1.05) | 0.96 (0.80-1.15) |
|  | ITT | 0.80 (0.68-0.94) | 1.04 (0.89-1.21) | 1.11 (1.00-1.24) |
| ***Safety*** |  |  |  |  |
| Safety composite | UT | 1.07 (0.80-1.44) | 1.10 (0.81-1.48) | 0.68 (0.53-0.88) |
|  | ITT | 1.05 (0.81-1.36) | 1.13 (0.86-1.48) | 0.72 (0.57-0.91) |
| Intracranial bleeding | UT | 0.69 (0.36-1.32) | 0.41 (0.17-0.97) | 0.69 (0.43-1.09) |
|  | ITT | 0.59 (0.31-1.12) | 0.49 (0.23-1.04) | 0.89 (0.59-1.32) |
| GI bleeding | UT | 1.31 (0.85-2.01) | 1.40 (0.90-2.16) | 0.77 (0.52-1.15) |
|  | ITT | 1.05 (0.70-1.58) | 1.23 (0.82-1.84) | 0.69 (0.48-0.99) |
| **Benefit/risk composite** ^†^ | UT | 0.80 (0.64-0.99) | 0.93 (0.75-1.14) | 0.84 (0.73-0.98) |
|  | ITT | 0.86 (0.74-0.99) | 1.08 (0.94-1.24) | 1.00 (0.91-1.10) |

^†^ benefit/risk composite: stroke/SE, all-cause mortality, and major bleeding.

**Supplemental Table S7.2.** Sensitivity analyses of comparative effectiveness and safety of each low-dose DOACs versus each other in an as-treated analysis and an intent-to-treat analysis, after IPTW.

|  | **Analysis** | **Hazard Ratio (95%CI)** | **Hazard Ratio**  **(95%CI)** | **Hazard Ratio**  **(95%CI)** |
| --- | --- | --- | --- | --- |
|  |  | **Dabigatran vs. apixaban** | **Rivaroxaban vs. apixaban** | **Dabigatran vs. rivaroxaban** |
| ***Effectiveness*** |  |  |  |  |
| Stroke (ischemic only)/SE | UT | 0.53 (0.30-0.93) | 0.70 (0.41-1.17) | 0.80 (0.40-1.59) |
|  | ITT | 0.55 (0.34-0.92) | 0.85 (0.54-1.34) | 0.70 (0.39-1.25) |
| All-cause mortality | UT | 0.43 (0.26-0.71) | 0.94 (0.65-1.35) | 0.68 (0.40-1.17) |
|  | ITT | 0.72 (0.59-0.87) | 1.02 (0.85-1.21) | 0.86 (0.67-1.09) |
| Effectiveness composite | UT | 0.49 (0.34-0.71) | 0.86 (0.64-1.17) | 0.75 (0.49-1.14) |
|  | ITT | 0.70 (0.59-0.84) | 1.00 (0.85-1.18) | 0.84 (0.67-1.05) |
| ***Safety*** |  |  |  |  |
| Safety composite | UT | 2.02 (1.42-2.86) | 1.58 (1.09-2.29) | 1.16 (0.79-1.72) |
|  | ITT | 1.79 (1.29-2.49) | 1.61 (1.14-2.27) | 1.07 (0.75-1.54) |
| Intracranial bleeding | UT | 1.35 (0.65-2.76) | 1.51 (0.75-3.05) | 1.70 (0.61-4.74) |
|  | ITT | 1.07 (0.54-2.12) | 1.36 (0.70-2.62) | 1.24 (0.50-3.08) |
| GI bleeding | UT | 2.47 (1.47-4.16) | 1.42 (0.77-2.60) | 1.26 (0.71-2.24) |
|  | ITT | 2.12 (1.31-3.45) | 1.61 (0.95-2.73) | 1.09 (0.65-1.84) |
| **Composite benefit/risk**^†^ | UT | 0.96 (0.75-1.22) | 1.06 (0.84-1.35) | 0.96 (0.72-1.28) |
|  | ITT | 0.85 (0.72-0.99) | 1.09 (0.94-1.27) | 0.88 (0.73-1.07) |

^†^ : benefit/risk composite: stroke/SE, all-cause mortality, and major bleeding.

**Supplemental Table S7.3.** Sensitivity analyses of comparative effectiveness and safety of each low-dose DOACs versus warfarin in an as-treated analysis of effectiveness and safety after IPTW including the year of base cohort entry*.

|  | **Analysis** | **Hazard Ratio (95%CI)** | **Hazard Ratio**  **(95%CI)** | **Hazard Ratio**  **(95%CI)** |
| --- | --- | --- | --- | --- |
|  |  | **Dabigatran vs. warfarin** | **Rivaroxaban vs. warfarin** | **Dabigatran vs.**  **warfarin** |
| ***Effectiveness*** |  |  |  |  |
| Stroke (ischemic only)/SE | UT | 0.85 (0.51-1.40) | 1.10 (0.69-1.75) | 1.24 (0.91-1.71) |
|  | UT* | 0.82 (0.49-1.37) | 1.06 (0.65-1.75) | 1.19 (0.83-1.73) |
| All-cause mortality | UT | 0.46 (0.30-0.70) | 0.65 (0.45-0.94) | 0.85 (0.68-1.06) |
|  | UT* | 0.46 (0.30-0.70) | 0.70 (0.48-1.02) | 0.98 (0.77-1.25) |
| Effectiveness composite | UT | 0.59 (0.42-0.81) | 0.78 (0.59-1.05) | 0.96 (0.80-1.15) |
|  | UT* | 0.58 (0.42-0.80) | 0.82 (0.61-1.10) | 1.04 (0.84-1.27) |
| ***Safety*** |  |  |  |  |
| Safety composite | UT | 1.07 (0.80-1.44) | 1.10 (0.81-1.48) | 0.68 (0.53-0.88) |
|  | UT* | 1.06 (0.79-1.42) | 1.35 (1.02-1.80) | 0.59 (0.42-0.79) |
| Intracranial bleeding | UT | 0.69 (0.36-1.32) | 0.41 (0.17-0.97) | 0.69 (0.43-1.09) |
|  | UT* | 0.67 (0.35-1.31) | 0.78 (0.40-1.53) | 0.53 (0.30-0.98) |
| GI bleeding | UT | 1.31 (0.85-2.01) | 1.40 (0.90-2.16) | 0.77 (0.52-1.15) |
|  | UT* | 1.30 (0.84-2.01) | 1.83 (1.21-2.75) | 0.54 (0.41-0.93) |
| **Composite benefit/risk**^†^ | UT | 0.80 (0.64-0.99) | 0.93 (0.75-1.14) | 0.84 (0.73-0.98) |
|  | UT* | 0.79 (0.63-0.98) | 1.06 (0.86-1.30) | 0.84 (0.71-1.00) |

^†^: benefit/risk composite: stroke/SE, all-cause mortality, and major bleeding.

**Supplemental Table S7.4.** Sensitivity analyses of comparative effectiveness and safety of each low-dose DOACs versus each other in an as-treated analysis of effectiveness and safety after IPTW including the year of base cohort entry*.

|  | **Analysis** | **Hazrad Ratio (95%CI)** | **Hazard Ratio**  **(95%CI)** | **Hazard Ratio**  **(95%CI)** |
| --- | --- | --- | --- | --- |
|  |  | **Dabigatran vs. warfarin** | **Rivaroxaban vs. warfarin** | **Dabigatran vs.**  **warfarin** |
| ***Effectiveness*** |  |  |  |  |
| Stroke (ischemic only)/SE | UT | 0.53 (0.30-0.93) | 0.70 (0.41-1.17) | 0.80 (0.40-1.59) |
|  | UT* | 0.42 (0.24-0.73) | 0.59 (0.34-1.02) | 0.58 (0.28-1.23) |
| All-cause mortality | UT | 0.43 (0.26-0.71) | 0.94 (0.65-1.35) | 0.68 (0.40-1.17) |
|  | UT* | 0.57 (0.39-0.85) | 1.02 (0.72-1.44) | 0.81 (0.48-1.34) |
| Effectiveness composite | UT | 0.49 (0.34-0.71) | 0.86 (0.64-1.17) | 0.75 (0.49-1.14) |
|  | UT* | 0.53 (0.39-0.74) | 0.88 (0.65-1.18) | 0.74 (0.48-1.13) |
| ***Safety*** |  |  |  |  |
| Safety composite | UT | 2.02 (1.42-2.86) | 1.58 (1.09-2.29) | 1.16 (0.79-1.72) |
|  | UT* | 1.85 (1.32-2.61) | 1.59 (1.10-2.30) | 1.07 (0.72-1.60) |
| Intracranial bleeding | UT | 1.35 (0.65-2.76) | 1.51 (0.75-3.05) | 1.70 (0.61-4.74) |
|  | UT* | 0.42 (0.16-1.14) | 0.98 (0.44-2.19) | 0.88 (0.30-2.63) |
| GI bleeding | UT | 2.47 (1.47-4.16) | 1.42 (0.77-2.60) | 1.26 (0.71-2.24) |
|  | UT* | 2.70 (1.61-4.53) | 1.66 (0.92-2.99) | 1.22 (0.69-2.15) |
| **Composite benefit/risk**^†^ | UT | 0.96 (0.75-1.22) | 1.06 (0.84-1.35) | 0.96 (0.72-1.28) |
|  | UT* | 0.94 (0.75-1.18) | 1.07 (0.85-1.35) | 0.91 (0.68-1.21) |

^†^ : benefit/risk composite: stroke/SE, all-cause mortality, and major bleeding.
