# Supplementary material for: Comparative Effectiveness and Safety of Low-Dose Oral Anticoagulants in Patients With Atrial Fibrillation
Source: Front Pharmacol. 2022 Jan 14;12:812018. doi: 10.3389/fphar.2021.812018 (PMC8795908; doi:10.3389/fphar.2021.812018)
Supplement: Supplementary file 4 [file Table9.docx]

**Supplementary Tables:**

**Supplemental Table S9.** Distribution of the follow-up time and the level of adherence at the 1-year follow-up visit.

|  | Distribution of follow-up time (days) | | Level of adherence (%) | |
| --- | --- | --- | --- | --- |
|  | Mean (95%CI) | Median [IQR] | Mean (95%CI) | Median [IQR] |
| **As-treated** |  |  |  |  |
| Dabigatran 110 mg | 250 (244-256) | 331 (118-365) | 68.4 (67.0-70.0) | 90.7 (32.3-100.0) |
| Rivaroxaban 15 mg | 255 (248-262) | 361 (117-365) | 69.9 (68.2-71.7) | 98.9 (32.2-100.0) |
| Apixaban 2.5 mg | 260 (256-265) | 361 (131-365) | 71.4 (70.2-72.5) | 98.9 (35.9-100.0) |
| Warfarin | 244 (241-246) | 333 (95-365) | 66.8 (66.1-67.4) | 91.1 (26.0-100.0) |
| **Intent-to-treat** |  |  |  |  |
| Dabigatran 110 mg | 310 (306-315) | 365 (365 -365) | 62.5 (60.8-64.1) | 74.2 (24.7-100.0) |
| Rivaroxaban 15 mg | 297 (291-302) | 365 (260-365) | 63.9 (62.1-65.7) | 78.9 (24.9-100.0) |
| Apixaban 2.5 mg | 290 (286-294) | 365 (230-365) | 64.2 (63.0-65.3) | 77.8 (26.8-100.0) |
| Warfarin | 296 (294-298) | 365 (263-365) | 56.7 (56.0-57.3) | 57.0 (15.6-100.0) |
