# Supplementary material for: Comparative Effectiveness and Safety of Low-Dose Oral Anticoagulants in Patients With Atrial Fibrillation
Source: Front Pharmacol. 2022 Jan 14;12:812018. doi: 10.3389/fphar.2021.812018 (PMC8795908; doi:10.3389/fphar.2021.812018)
Supplement: Supplementary file 5 [file Table2.docx]

**Supplementary Tables:**

**Supplemental Table S2.** Definitions of the CHA_2_DS_2_-VASc and Modified HAS-BLED risk scores.

| **Risk score definition** | Points if present |
| --- | --- |
|  |  |
| **CHA_2_DS_2_-VASc** |  |
| Congestive heart failure or left ventricular dysfunction | 1 |
| Hypertension | 1 |
| Age 65 – 74 years | 1 |
| Age ≥ 75 years | 2 |
| Diabetes mellitus | 1 |
| Stroke (ischemic stroke, transient ischemic disease or systemic embolism) | 2 |
| Vascular disease (myocardial infarction, peripheral arterial disease or aortic plaque) | 1 |
| Sex category (female) | 1 |
|  |  |
| **HAS-BLED** |  |
| Hypertension | 1 |
| Abnormal renal function | 1 |
| Abnormal hepatic function |  |
| Abnormal stroke (ischemic stroke, transient ischemic disease) | 1 |
| Bleeding | 1 |
| Older than 65 | 1 |
| Labile international normalized ratio (not available) | 1 |
| Drugs (ASA, clopidogrel, prasugrel, ticagrelor, ticlopidine, or non-steroidal anti-inflammatory drugs) in the 1 month preceding the ICH hospitalization or 1 month after discharge | 1 |
| Alcohol intake | 1 |
