# Supplementary material for: Comparative Effectiveness and Safety of Low-Dose Oral Anticoagulants in Patients With Atrial Fibrillation
Source: Front Pharmacol. 2022 Jan 14;12:812018. doi: 10.3389/fphar.2021.812018 (PMC8795908; doi:10.3389/fphar.2021.812018)
Supplement: Supplementary file 6 [file Table5.docx]

**Supplementary Tables:**

**Supplemental Table S5.1.** The initial cohort vs. the cohort after IPTW, for the dabigatran 110 mg and warfarin groups.

|  | **Initial Cohort** | | | **Cohort After IPTW** | | |
| --- | --- | --- | --- | --- | --- | --- |
|  | Dabigatran  (n=1,929) | Warfarin  (n=14,700) | Absolute standardized difference | Dabigatran  (n=1,929) | Warfarin  (n=14,700) | Absolute standardized difference |
| **Age** – mean (SD) | 81.2 (7.3) | 80.1 (9.1) | 0.15 | 80.2 (7.7) | 80.2 (9.1) | 0.01 |
| **Female** sex | 57.9% | 55.5% | 0.05 | 56.8% | 55.8% | 0.02 |
| **CHA2DS2-VAS Score** including the index hospitalization and 3 years prior to the index claim | 3.9 (1.3) | 4.0 (1.4) | 0.08 | 4.0 (1.3) | 3.9 (1.4) | 0.01 |
| **HAS-BLED Score** including the index hospitalization and 3 years prior to the index claim | 3.0 (1.2) | 3.3 (1.3) | 0.24 | 3.3 (1.2) | 3.3 (1.3) | <0.01 |
| **Charlson Score Index (**including the index hospitalization and 3 years prior to the index claim) | 4.2 (3.4) | 5.0 (3.4) | 0.24 | 4.9 (3.5) | 4.9 (3.4) | 0.02 |
| **Frailty Score** | 12.0 (6.8) | **12.6 (7.1)** | 0.10 | 12.7 (6.9) | 12.6 (7.0) | 0.02 |
| **Comorbidities** (the index hospitalization and 3 years prior to the index claim)^¥^ - no (%) |  |  |  |  |  |  |
| Hypertension | 82.6 % | 84.9% | 0.06 | 84.4 % | 84.6% | <0.01 |
| Dyslipidemia | 50.1% | 53.8% | 0.07 | 51.8% | 53.4% | 0.03 |
| Diabetes | 30.6% | 38.8% | 0.17 | 35.0% | 37.9% | 0.06 |
| Coronary artery disease | 51.8% | 60.0% | 0.17 | 59.3% | 59.1% | <0.01 |
| Acute myocardial infarction | 9.6% | 15.7% | 0.18 | 14.0% | 15.0% | 0.03 |
| Chronic heart failure | 35.3% | 44.1% | 0.18 | 41.2% | 43.1% | 0.04 |
| Cardiomyopathy | 5.0% | 6.4% | 0.06 | 5.6% | 6.2% | 0.03 |
| Other cardiac rhythm disorders | 23.2% | 20.4% | 0.07 | 20.3% | 20.7% | 0.01 |
| Valvular disease | 23.0% | 16.8% | 0.15 | 22.3% | 22.3% | <0.01 |
| Prior cerebrovascular disease including TIA | 22.9% | 21.2% | 0.04 | 21.5% | 21.4% | <0.01 |
| Prior ischemic stroke | 23.0% | 21.0% | 0.04 | 21.3% | 21.1% | <0.01 |
| Peripheral vascular disease | 18.7% | 25.1% | 0.15 | 23.3% | 24.4% | 0.02 |
| Chronic renal failure | 25.3% | 45.6% | 0.44 | 42.6% | 43.3% | 0.01 |
| Chronic renal failure < 30 mL/min | 1.4% | 8.2% | 0.32 | 5.7% | 7.4% | 0.07 |
| Acute renal failure | 12.8% | 29.5% | 0.42 | 25.1% | 27.6% | 0.06 |
| Chronic obstructive pulmonary disease/asthma | 34.7% | 39.0% | 0.09 | 37.6% | 38.5% | 0.02 |
| Liver disease | 1.9% | 2.3% | 0.03 | 2.2% | 2.2% | <0.01 |
| Systemic embolism | 1.5% | 3.1% | 0.11 | 2.8% | 3.0% | 0.01 |
| Depression | 13.6% | 11.3% | 0.07 | 11.8% | 11.5% | 0.01 |
| Hypothyroidism | 22.1% | 24.7% | 0.06 | 24.4% | 24.4% | <0.01 |
| Neurologic disorder | 26.4% | 25.3% | 0.03 | 26.2% | 25.4% | 0.02 |
| Major bleeding | 27.7% | 33.0% | 0.12 | 31.8% | 32.4% | 0.01 |
| Malignant cancer | 24.7% | 24.3% | 0.01 | 24.2% | 24.4% | 0.01 |
| **Medical procedures (in the 3 years prior to the index claim)** |  |  |  |  |  |  |
| Cardiac catheterization | 3.1% | 4.0% | 0.05 | 3.5% | 3.8% | 0.02 |
| Percutaneous coronary intervention - stent | 2.1% | 3.0% | 0.06 | 3.6% | 2.9% | 0.04 |
| Coronary artery bypass grafting | 0.6% | 0.5% | 0.02 | 1.0% | 0.7% | 0.03 |
| Implantable cardiac device | 0.04% | 0.0% | 0.01 | 0.8% | 0.5% | 0.04 |
| **Medications in the 2 weeks prior to the index claim** |  |  |  |  |  |  |
| Diuretics | 36.7 % | 44.3 % | 0.16 | 42.3% | 43.4% | 0.02 |
| Loop diuretics | 28.5 % | 37.2 % | 0.19 | 35.2% | 36.2% | 0.02 |
| B-blockers | 62.2 % | 62.3 % | <0.01 | 60.8% | 62.2% | 0.03 |
| Renin-angiotensin system Inhibitors | 41.1 % | 37.8 % | 0.07 | 38.9% | 38.2% | 0.02 |
| Calcium channel blockers | 37.1% | 40.0% | 0.06 | 39.1% | 39.6% | 0.01 |
| Statin | 43.8% | 47.9% | 0.08 | 46.8% | 47.4% | 0.01 |
| Antidiabetics | 18.1 % | 23.0 % | 0.12 | 20.8% | 22.4% | 0.04 |
| Antiplatelet agents (excluding low-dose ASA) | 4.8% | 6.2% | 0.06 | 6.2% | 6.0% | 0.01 |
| Low dose ASA | 27.9% | 31.9% | 0.09 | 31.8% | 31.5% | 0.01 |
| Proton pump inhibitors | 42.8% | 46.1% | 0.07 | 46.1% | 45.8% | 0.01 |
| NSAIDs | 1.6% | 1.4% | 0.02 | 1.4% | 1.4% | <0.01 |
| Amiodarone or propafenone | 10.0% | 10.1% | <0.01 | 9.9% | 10.1% | 0.01 |
| Digoxin | 13.9% | 13.4% | 0.01 | 14.6% | 13.5% | 0.03 |
| Antidepressant  SSRIs: citalopram, escitalopram, fluoxetine, paroxetine, sertraline | 9.1% | 8.6% | 0.02 | 9.0% | 8.7% | 0.01 |
| PGP inhibitor use | 58.0% | 62.1% | 0.08 | 61.0% | 61.6% | 0.01 |
| Number of distinct AHFS classes, mean (SD) | 8.4 (4.1) | 9.2 (4.5) | 0.18 | 9.1 (4.3) | 9.1 (4.5) | <0.01 |
| **Medical service consumption in the year prior to the index claim** |  |  |  |  |  |  |
| Number of visits to a specialist, mean (SD) | 1.4 (3.0) | 1.2 (2.1) | 0.09 | 1.3 (3.0) | 1.3 (3.0) | 0.03 |
| Number of family physician visits, mean (SD) | 1.5 (3.2) | 1.3 (3.0) | 0.06 | 1.3 (2.2) | 1.2 (2.3) | 0.02 |
| Number of emergency room visits, mean (SD) | 3.2 (2.8) | 3.2 (2.8) | 0.01 | 3.3 (2.8) | 3.2 (2.8) | 0.02 |
| **Hospital service consumption in the 3 years prior to the index claim** |  |  |  |  |  |  |
| Mean number of all-cause hospital admission | 2.3 (1.7) | 2.4 (1.8) | 0.03 | 2.4 (1.7) | 2.4 (1.8) | 0.01 |
| Length of stay | 9.1 (11.0) | 11.0 (12.2) | 0.12 | 11.1 (14.2) | 10.8 (12.0) | 0.03 |

References used for the propensity score: 1) Mentias A et al., JAMA Network Open 2018; 1:e182870; 2) Lopes et al. Am J Med 2018`; IPTW: inverse probability of treatment weighting

**Supplemental Table S5.2.** The initial cohort vs. the cohort after IPTW, for the rivaroxaban 15 mg and warfarin groups.

|  | **Initial Cohort** | | | | **Cohort After IPTW** | | | |
| --- | --- | --- | --- | --- | --- | --- | --- | --- |
|  | Rivaroxaban  (n=1,718) | Warfarin  (n=14,700) | Absolute standardized difference | Rivaroxaban  (n=1,718) | | Warfarin  (n=14,700) | Absolute standardized difference |  |
| **Age** – mean (SD) | 82.9 (7.2) | 80.1 (9.1) | 0.38 | 80.7 (7.8) | | 80.4 (9.1) | 0.04 |  |
| **Female** sex | 61.3% | 55.5% | 0.12 | 57.0% | | 56.1% | 0.02 |  |
| **CHA2DS2-VAS Score** including the index hospitalization and 3 years prior to the index claim | 4.0 (1.3) | 4.0 (1.4) | 0.03 | 4.0 (1.3) | | 4.0 (1.4) | 0.05 |  |
| **HAS-BLED Score** including the index hospitalization and 3 years prior to the index claim | 3.2 (1.3) | 3.3 (1.3) | 0.12 | 3.4(1.3) | | 3.3 (1.3) | 0.05 |  |
| **Charlson Score Index (**including the index hospitalization and 3 years prior to the index claim) | 4.7 (3.5) | 5.0 (3.4) | 0.11 | 5.2 (3.7) | | 5.0 (3.4) | 0.07 |  |
| **Frailty Score** | 12.5 (6.8) | 12.6 (7.1) | 0.02 | 12.9 (6.9) | | 12.6 (7.0) | 0.04 |  |
| **Comorbidities** (the index hospitalization and 3 years prior to the index claim)^¥^ - no (%) |  |  |  |  | |  |  |  |
| Hypertension | 84.0% | 84.9% | 0.02 | 86.0 % | | 84.8% | 0.03 |  |
| Dyslipidemia | 50.5% | 53.8% | 0.07 | 53.2% | | 53.5% | <0.01 |  |
| Diabetes | 31.1% | 38.8% | 0.02 | 38.8% | | 38.0% | 0.03 |  |
| Coronary artery disease | 53.8% | 60.0% | 0.01 | 60.3% | | 59.4% | 0.02 |  |
| Acute myocardial infarction | 14.4% | 15.7% | 0.03 | 16.5% | | 15.6% | 0.02 |  |
| Chronic heart failure | 39.2% | 44.1% | 0.10 | 44.5% | | 43.6% | 0.02 |  |
| Cardiomyopathy | 5.6% | 6.4% | 0.03 | 6.2% | | 6.3% | 0.01 |  |
| Other cardiac rhythm disorders | 18.0% | 20.4% | 0.06 | 20.2% | | 20.2% | <0.01 |  |
| Valvular disease | 19.4% | 23.0% | 0.09 | 22.0% | | 22.6% | 0.01 |  |
| Prior cerebrovascular disease including TIA | 18.4% | 21.2% | 0.07 | 20.7% | | 20.9% | <0.01 |  |
| Prior ischemic stroke | 17.8% | 13.6% | 0.08 | 20.5% | | 20.6% | <0.01 |  |
| Peripheral vascular disease | 21.3% | 25.1% | 0.09 | 25.6% | | 24,7% | 0.02 |  |
| Chronic renal failure | 40.4% | 45.6% | 0.11 | 49.0% | | 45.1% | 0.08 |  |
| Chronic renal failure < 30 mL/min | 3.0% | 8.2% | 0.23 | 7.2% | | 7.6% | 0.02 |  |
| Acute renal failure | 24.9% | 29.5% | 0.11 | 30.9% | | 29.1% | 0.04 |  |
| Chronic obstructive pulmonary disease/asthma | 37.7% | 40.0% | 0.03 | 40.5% | | 38.8% | 0.03 |  |
| Liver disease | 1.9% | 2.3% | 0.02 | 2.6% | | 2.2% | 0.03 |  |
| Systemic embolism | 2.1% | 3.1% | 0.07 | 3.0% | | 3.0% | <0.01 |  |
| Depression | 12.6% | 11.3% | 0.04 | 11.0% | | 11.4% | 0.01 |  |
| Hypothyroidism | 27.1% | 24.7% | 0.06 | 26.0% | | 25.0% | 0.02 |  |
| Neurologic disorder | 27.3% | 25.3% | 0.05 | 25.2% | | 25.5% | 0.01 |  |
| Major bleeding | 30.7% | 33.0% | 0.05 | 34.8% | | 32.8% | 0.04 |  |
| Malignant cancer | 25.1% | 24.3% | 0.02 | 26.1% | | 24.4% | 0.04 |  |
| **Medical procedures (in the 3 years prior to the index claim)** |  |  |  |  | |  |  |  |
| Cardiac catheterization | 2.7% | 4.0% | 0.07 | 3.9% | | 3.8% | <0.01 |  |
| Percutaneous coronary intervention - stent | 2.7% | 3.0% | 0.01 | 3.3% | | 3.0% | 0.02 |  |
| Coronary artery bypass grafting | 0.6% | 0.8% | 0.02 | 0.9% | | 0.7% | 0.02 |  |
| Implantable cardiac device | 0.06% | 0.5% | 0.08 | 0.1% | | 0.4% | 0.06 |  |
| **Medications in the 2 weeks prior to the index claim** |  |  |  |  | |  |  |  |
| Diuretics | 41.5 % | 44.3 % | 0.06 | 45.6 % | | 44.1% | 0.03 |  |
| Loop diuretics | 33.2 % | 37.2 % | 0.08 | 38.6 % | | 36.8% | 0.04 |  |
| B-blockers | 64.0 % | 62.3 % | 0.04 | 62.2 % | | 62.4% | 0.01 |  |
| Renin-angiotensin system Inhibitors | 37.1 % | 37.8 % | 0.02 | 38.3 % | | 37.8% | 0.01 |  |
| Calcium channel blockers | 38.4 % | 40.0 % | 0.03 | 39.7 % | | 39.8% | <0.01 |  |
| Statin | 42.0% | 47.8% | 0.12 | 47.5% | | 47.3% | <0.01 |  |
| Antidiabetics | 17.4 % | 23.0 % | 0.14 | 23.4 % | | 22.5% | 0.02 |  |
| Antiplatelet agents (excluding low-dose ASA) | 5.4% | 6.2% | 0.03 | 6.3% | | 6.1% | 0.01 |  |
| Low dose ASA | 26.9% | 31.9% | 0.11 | 31.3% | | 31.4% | <0.01 |  |
| Proton pump inhibitors | 42.4% | 46.1% | 0.07 | 46.2% | | 45.7% | 0.01 |  |
| NSAIDs | 1.1% | 1.4% | 0.03 | 1.3% | | 1.3% | <0.01 |  |
| Amiodarone or propafenone | 9.6% | 10.1% | 0.02 | 10.4% | | 10.1% | 0.01 |  |
| Digoxin | 11.6% | 13.4% | 0.05 | 12.9% | | 13.3% | 0.01 |  |
| Antidepressant  SSRIs: citalopram, escitalopram, fluoxetine, paroxetine, sertraline | 9.2% | 8.6% | 0.02 | 8.5% | | 8.7% | 0.01 |  |
| P-Glycoprotein inhibitor use | 60.4% | 62.1% | 0.03 | 61.9% | | 61.9% | <0.01 |  |
| Number of distinct AHFS classes, mean (SD) | 8.8 (4.2) | 9.2 (4.5) | 0.07 | 9.3 (4.4) | | 9.1 (4.5) | 0.03 |  |
| **Medical service consumption in the year prior to the index claim** |  |  |  |  | |  |  |  |
| Number of visits to a specialist, mean (SD) | 1.2 (2.1) | 1.2 (2.1) | 0.02 | 1.3 (2.9) | | 1.3 (3.0) | 0.01 |  |
| Number of family physician visits, mean (SD) | 1.4 (3.0) | 1.3 (3.0) | 0.03 | 1.2 (2.0) | | 1.2 (2.1) | 0.01 |  |
| Number of emergency room visits, mean (SD) | 3.3 (2.5) | 3.2 (2.8) | 0.02 | 3.3 (2.6) | | 3.2 (2.8) | 0.03 |  |
| **Hospital service consumption in the 3 years prior to the index claim** |  |  |  |  | |  |  |  |
| Mean number of all-cause hospital admission | 2.2 (1.7) | 2.4 (1.8) | 0.07 | 2.4 (2.0) | | 2.4 (1.8) | 0.04 |  |
| Length of stay | 9.7 (11.0) | 11.0 (12.2) | 0.11 | 11.1 (13.4) | | 10.8 (12.0) | 0.02 |  |

References used for the propensity score: 1) Mentias A et al., JAMA Network Open 2018; 1:e182870; 2) Lopes et al. Am J Med 2018; IPTW: inverse probability of treatment weighting.

|  |  |  |  |  |  |  |
| --- | --- | --- | --- | --- | --- | --- |

**Supplemental Table S5.3.** The initial cohort vs. the cohort after IPTW, for the apixaban 2.5 mg and warfarin groups.

|  | **Initial Cohort** | | | | **Cohort After IPTW** | | | |
| --- | --- | --- | --- | --- | --- | --- | --- | --- |
|  | Apixaban  (n=3,829) | Warfarin  (n=14,700) | Absolute standardized difference | Apixaban  (n=3,829) | | Warfarin  (n=14,700) | Absolute standardized difference |  |
| **Age** – mean (SD) | 86.4 (6.1) | 80.1 (9.1) | 1.04 | 82.2 (7.9) | | 81.5 (9.1) | 0.09 |  |
| **Female** sex | 68.5% | 55.5% | 0.27 | 58.9% | | 58.2% | 0.01 |  |
| **CHA2DS2-VAS Score** including the index hospitalization and 3 years prior to the index claim | 4.3 (1.2) | 4.0 (1.4) | 0.25 | 4.2 (1.3) | | 4.0 (1.4) | 0.09 |  |
| **HAS-BLED Score** including the index hospitalization and 3 years prior to the index claim | 3.3 (1.3) | 3.3 (1.3) | 0.04 | 3.4 (1.3) | | 3.3 (1.3) | 0.09 |  |
| **Charlson Score Index (**including the index hospitalization and 3 years prior to the index claim) | 4.8 (3.3) | 5.0 (3.4) | 0.07 | 5.3 (3.5) | | 5.0 (3.4) | 0.08 |  |
| **Frailty Score** | 13.6 (6.9) | 12.6 (7.1) | 0.13 | 13.3 (6.9) | | 12.9 (7.1) | 0.06 |  |
| **Comorbidities** (the index hospitalization and 3 years prior to the index claim)^¥^ - no (%) |  |  |  |  | |  |  |  |
| Hypertension | 83.8 % | 84.9% | 0.03 | 86.1 % | | 84.7% | 0.04 |  |
| Dyslipidemia | 49.0% | 53.8% | 0.10 | 53.2% | | 52.7% | 0.01 |  |
| Diabetes | 28.8% | 38.8% | 0.21 | 37.7% | | 36.8% | 0.02 |  |
| Coronary artery disease | 53.5% | 60.0% | 0.13 | 59.8% | | 58.8% | 0.02 |  |
| Acute myocardial infarction | 16.5% | 15.7% | 0.03 | 17.1% | | 15.9% | 0.03 |  |
| Chronic heart failure | 42.7% | 44.1% | 0.03 | 46.1% | | 44.0% | 0.04 |  |
| Cardiomyopathy | 5.0% | 6.4% | 0.06 | 5.7% | | 6.1% | 0.02 |  |
| Other cardiac rhythm disorders | 18.9% | 20.4% | 0.04 | 19.7% | | 20.1% | 0.01 |  |
| Valvular disease | 21.9% | 23.0% | 0.03 | 23.2% | | 22.8% | 0.01 |  |
| Prior cerebrovascular disease including TIA | 19.4% | 21.2% | 0.04 | 22.1% | | 20.8% | 0.03 |  |
| Prior ischemic stroke | 18.6% | 21.0% | 0.06 | 21.8% | | 20.4% | 0.03 |  |
| Peripheral vascular disease | 21.4% | 25.1% | 0.09 | 25.9% | | 24.4% | 0.04 |  |
| Chronic renal failure | 45.3% | 45.6% | 0.01 | 51.3% | | 45.9% | 0.10 |  |
| Chronic renal failure < 30 mL/min | 3.6% | 8.2% | 0.20 | 8.6% | | 7.2% | 0.05 |  |
| Acute renal failure | 28.1% | 29.5% | 0.03 | 33.6% | | 29.5% | 0.09 |  |
| Chronic obstructive pulmonary disease/asthma | 34.3% | 39.0% | 0.10 | 37.6% | | 37.9% | 0.01 |  |
| Liver disease | 1.5% | 2.3% | 0.05 | 2.4% | | 2.1% | 0.02 |  |
| Systemic embolism | 1.9% | 3.1% | 0.08 | 3.2% | | 2.9% | 0.02 |  |
| Depression | 12.3% | 11.3% | 0.03 | 11.3% | | 11.5% | 0.01 |  |
| Hypothyroidism | 28.3% | 24.7% | 0.08 | 26.4% | | 25.4% | 0.02 |  |
| Neurologic disorder | 31.1% | 25.3% | 0.13 | 27.5% | | 26.5% | 0.02 |  |
| Major bleeding | 33.9% | 33.0% | 0.02 | 36.1% | | 33.3% | 0.06 |  |
| Malignant cancer | 25.1% | 24.3% | 0.02 | 25.5% | | 24.6% | 0.02 |  |
| **Medical procedures (in the 3 years prior to the index claim)** |  |  |  |  | |  |  |  |
| Cardiac catheterization | 2.6% | 4.0% | 0.08 | 3.8% | | 3.7% | 0.01 |  |
| Percutaneous coronary intervention - stent | 2.3% | 3.0% | 0.05 | 2.9% | | 2.8% | <0.01 |  |
| Coronary artery bypass grafting | 0.2% | 0.8% | 0.08 | 0.5% | | 0.6% | 0.01 |  |
| Implantable cardiac device | 0.0% | 0.5% | 0.10 | 0.0% | | 0.4% | 0.09 |  |
| **Medications in the 2 weeks prior to the index claim** |  |  |  |  | |  |  |  |
| Diuretics | 42.6 % | 44.3 % | 0.03 | 46.1 % | | 44.2% | 0.04 |  |
| Loop diuretics | 36.5 % | 37.2 % | 0.02 | 39.2 % | | 37.3% | 0.04 |  |
| B-blockers | 65.5 % | 62.3 % | 0.07 | 61.2 % | | 62.9% | 0.03 |  |
| Renin-angiotensin system Inhibitors | 35.2 % | 37.8 % | 0.06 | 37.3 % | | 37.3% | <0.01 |  |
| Calcium channel blockers | 39.5 % | 40.0 % | 0.01 | 40.8 % | | 39.9% | 0.02 |  |
| Statin | 41.3% | 47.9% | 0.13 | 46.0% | | 46.4% | 0.01 |  |
| Antidiabetics | 16.7 % | 23.0 % | 0.16 | 21.9 % | | 21.7% | <0.01 |  |
| Antiplatelet agents (excluding low-dose ASA) | 5.9% | 6.2% | 0.01 | 6.2% | | 6.1% | <0.01 |  |
| Low dose ASA | 26.5% | 31.9% | 0.12 | 30.9% | | 30.8% | <0.01 |  |
| Proton pump inhibitors | 44.0% | 46.1% | 0.04 | 47.0% | | 45.7% | 0.03 |  |
| NSAIDs | 1.1% | 1.4% | 0.02 | 1.3% | | 1.3% | <0.01 |  |
| Amiodarone or propafenone | 10.1% | 10.1% | <0.01 | 9.7% | | 10.1% | 0.01 |  |
| Digoxin | 10.2% | 13.4% | 0.10 | 12.3% | | 12.8% | 0.01 |  |
| Antidepressant  SSRIs: citalopram, escitalopram, fluoxetine, paroxetine, sertraline | 10.0% | 8.6% | 0.05 | 8.5% | | 8.8% | 0.01 |  |
| PGP inhibitor use | 60.4% | 62.1% | 0.03 | 62.1% | | 61.7% | 0.01 |  |
| Number of distinct AHFS classes, mean (SD) | 9.1 (4.1) | 9.2 (4.5) | 0.01 | 9.2 (4.2) | | 9.2 (4.4) | 0.02 |  |
| **Medical service consumption in the year prior to the index claim** |  |  |  |  | |  |  |  |
| Number of visits to a specialist, mean (SD) | 1.3 (2.7) | 1.2 (2.1) | 0.07 | 1.3 (2.8) | | 1.3 (3.0) | 0.01 |  |
| Number of family physician visits, mean (SD) | 1.4 (3.0) | 1.3 (3.0) | 0.01 | 1.2 (2.2) | | 1.2 (2.4) | 0.01 |  |
| Number of emergency room visits, mean (SD) | 3.3 (2.6) | 3.2 (2.8) | 0.02 | 3.3 (2.6) | | 3.2 (2.8) | 0.02 |  |
| **Hospital service consumption in the 3 years prior to the index claim** |  |  |  |  | |  |  |  |
| Mean number of all-cause hospital admission | 2.2 (1.6) | 2.4 (1.8) | 0.10 | 2.4 (1.9) | | 2.4 (1.9) | 0.05 |  |
| Length of stay | 10.5 (11.6) | 11.0 (12.2) | 0.03 | 11.2 (13.2) | | 11.2 (13.2) | 0.02 |  |

References used for the propensity score: 1) Mentias A et al., JAMA Network Open 2018; 1:e182870; 2) Lopes et al. Am J Med 2018; IPTW: inverse probability of treatment weighting.

**Supplemental Table S5.4.** The initial cohort vs. the cohort after IPTW, for the dabigatran 110 mg and apixaban 2.5 mg groups.

|  | **Initial Cohort** | | | **Cohort After IPTW** | | |
| --- | --- | --- | --- | --- | --- | --- |
|  | Dabigatran  (n=1,929) | Apixaban  (n=3,829) | Absolute standardized difference | Dabigatran  (n=1,929) | Apixaban  (n=3,829) | Absolute standardized difference |
| **Age** – mean (SD) | 81.2 (7.3) | 86.4 (6.1) | 0.72 | 84.2 (6.6) | 84.2 (7.8) | 0.01 |
| **Female** sex | 57.9% | 68.5% | 0.22 | 64.5% | 64.9% | 0.01 |
| **CHA2DS2-VAS Score** including the index hospitalization and 3 years prior to the index claim | 3.9 (1.3) | 4.3 (1.2) | 0.35 | 4.1 (1.2) | 4.1 (1.3) | 0.01 |
| **HAS-BLED Score** including the index hospitalization and 3 years prior to the index claim | 3.0 (1.2) | 3.3 (1.3) | 0.19 | 3.2 (1.2) | 3.2 (1.3) | 0.03 |
| **Charlson Score Index (**including the index hospitalization and 3 years prior to the index claim) | 4.2 (3.4) | 4.8 (3.3) | 0.16 | 4.6 (3.4) | 4.6 (3.3) | 0.02 |
| **Frailty score** | 12.0 (6.8) | 13.6 (6.9) | 0.23 | 13.1 (6.8) | 13.0 (7.0) | 0.02 |
| **Comorbidities** (the index hospitalization and 3 years prior to the index claim)^¥^ - no (%) |  |  |  |  |  |  |
| Hypertension | 82.6 % | 83.8 % | 0.03 | 83.4 % | 83.1% | 0.01 |
| Dyslipidemia | 50.1% | 49.0% | 0.02 | 47.9% | 49.2% | 0.02 |
| Diabetes | 30.6% | 28.8% | 0.04 | 28.7% | 29.4% | 0.02 |
| Coronary artery disease | 51.8% | 53.5% | 0.03 | 52.4% | 52.7% | 0.01 |
| Acute myocardial infarction | 9.6% | 16.5% | 0.20 | 13.9% | 14.4% | 0.02 |
| Chronic heart failure | 35.3% | 42.7% | 0.15 | 40.2% | 40.4% | <0.01 |
| Cardiomyopathy | 5.0% | 5.0% | <0.01 | 4.5% | 4.9% | 0.02 |
| Other cardiac rhythm disorders | 23.2% | 18.9% | 0.10 | 20.1% | 19.9% | 0.01 |
| Valvular disease | 16.8% | 21.9% | 0.13 | 20.6% | 20.5% | <0.01 |
| Prior cerebrovascular disease including TIA | 22.9% | 19.4% | 0.09 | 21.6% | 20.5% | 0.03 |
| Prior ischemic stroke | 22.6% | 18.6% | 0.10 | 21.1% | 19.9% | 0.03 |
| Peripheral vascular disease | 18.7% | 21.4% | 0.07 | 20.3% | 20.2% | <0.01 |
| Chronic renal failure | 25.3% | 45.3% | 0.43 | 39.3% | 38.3% | 0.02 |
| Chronic renal failure <30 mL/min | 1.4% | 3.6% | 0.14 | 3.4% | 2.8% | 0.03 |
| Acute renal failure | 12.8% | 28.1% | 0.39 | 23.5% | 23.0% | 0.01 |
| Chronic obstructive pulmonary disease/asthma | 34.7% | 34.3% | 0.01 | 35.1% | 34.4% | 0.02 |
| Liver disease | 1.9% | 1.5% | 0.03 | 1.8% | 1.8% | <0.01 |
| Systemic embolism | 1.5% | 1.9% | 0.03 | 1.9% | 1.8% | <0.01 |
| Depression | 13.6% | 12.3% | 0.04 | 12.6% | 12.8% | <0.01 |
| Hypothyroidism | 22.1% | 28.3% | 0.14 | 25.1% | 26.1% | 0.02 |
| Neurologic disorder | 26.4% | 31.1% | 0.10 | 30.3% | 29.4% | 0.02 |
| Prior major bleeding | 27.7% | 33.9% | 0.13 | 33.5% | 32.3% | 0.03 |
| Malignant cancer | 24.7% | 25.1% | 0.01 | 25.6% | 24.8% | 0.02 |
| **Medical procedures (in the 3 years prior to the index claim)** |  |  |  |  |  |  |
| Cardiac catheterization | 3.1% | 2.6% | 0.03 | 2.9% | 2.7% | 0.01 |
| Percutaneous coronary intervention - stent | 2.1% | 2.3% | 0.01 | 2.3% | 2.3% | <0.01 |
| Coronary artery bypass grafting | 0.6% | 0.2% | 0.06 | 0.3% | 0.2% | 0.01 |
| Implantable cardiac device | 0.6% | 0.0% | 0.11 | 0.2% | 0.0% | 0.06 |
| **Medications in the 2 weeks prior to the index claim** |  |  |  |  |  |  |
| Diuretics | 36.7% | 42.6% | 0.12 | 41.0% | 40.7% | <0.01 |
| Loop diuretics | 28.5% | 36.5% | 0.17 | 34.4% | 34.1% | 0.01 |
| B-blockers | 62.2% | 65.5% | 0.07 | 63.3% | 63.4% | <0.01 |
| Renin-angiotensin system Inhibitors | 41.1% | 35.2% | 0.12 | 38.4% | 37.0% | 0.03 |
| Calcium channel blockers | 37.1% | 39.5% | 0.05 | 39.6% | 38.8% | 0.02 |
| Statin | 43.8% | 41.3% | 0.05 | 41.0% | 41.9% | 0.02 |
| Antidiabetics | 18.1% | 16.7% | 0.04 | 16.7% | 17.2% | 0.01 |
| Antiplatelet agents (excluding low-dose ASA) | 4.8% | 5.9% | 0.05 | 6.0% | 5.5% | 0.02 |
| Low-dose ASA | 27.9% | 26.5% | 0.03 | 28.3% | 26.9% | 0.03 |
| Proton pump inhibitors | 42.8% | 44.0% | 0.02 | 43.7% | 43.8% | <0.01 |
| NSAIDs | 1.6% | 1.1% | 0.04 | 1.3% | 1.3% | <0.01 |
| Amiodarone | 10.0% | 10.1% | <0.01 | 10.4% | 9.7% | 0.03 |
| Digoxin | 13.9% | 10.2% | 0.11 | 11.9% | 11.6% | 0.01 |
| Antidepressant | 9.1% | 10.0% | 0.03 | 10.5% | 10.1% | 0.01 |
| PGP inhibitor use | 58.0% | 60.4% | 0.05 | 59.4% | 59.8% | 0.01 |
| Number of medications | 8.4 (4.1) | 9.1 (4.1) | 0.19 | 9.0 (4.2) | 8.9 (4.1) | 0.03 |
| **Medical service consumption in the year prior to the index claim** |  |  |  |  |  |  |
| Number of visits to a specialist, mean (SD) | 1.4 (3.0) | 1.3 (2.7) | 0.03 | 1.3 (3.0) | 1.3 (3.0) | 0.01 |
| Number of family physician visits, mean (SD) | 1.5 (3.2) | 1.3 (3.0) | 0.05 | 1.4 (2.9) | 1.3 (2.8) | 0.01 |
| Number of emergency room visits, mean (SD) | 3.2 (2.8) | 3.3 (2.6) | 0.03 | 3.2 (2.6) | 3.3 (2.7) | 0.01 |
| **Hospital service consumption in the 3 years prior to the index claim** |  |  |  |  |  |  |
| Mean number of all-cause hospital admission | 2.3 (1.7) | 2.2 (1.6) | 0.08 | 2.3 (1.5) | 2.3 (1.7) | 0.01 |
| Length of stay | 9.1 (11.0) | 10.5 (11.6) | 0.13 | 10.1 (10.9) | 10.0 (11.3) | 0.01 |

References used for the propensity score: 1) Mentias A et al., JAMA Network Open 2018; 1:e182870; 2) Lopes et al. Am J Med 2018; IPTW: inverse probability of treatment weighting.

**Supplemental Table S5.5.** The initial cohort vs. the cohort after IPTW, for the rivaroxaban 15 mg and apixaban 2.5 mg.

|  | **Initial Cohort** | | | **Cohort After IPTW** | | | |
| --- | --- | --- | --- | --- | --- | --- | --- |
|  | Rivaroxaban  (n=1,718) | Apixaban  (n=3,829) | Absolute standardized difference | Rivaroxaban  (n=1,718) | Apixaban  (n=3,829) | Absolute standardized difference |  |
| **Age** – mean (SD) | 82.9 (7.2) | 86.4 (6.1) | 0.49 | 85.3 (6.7) | 85.2 (7.0) | 0.01 |  |
| **Female** sex | 61.3% | 68.5% | 0.15 | 65.8% | 66.1% | 0.01 |  |
| **CHA2DS2-VAS Score** including the index hospitalization and 3 years prior to the index claim | 4.0 (1.3) | 4.3 (1.2) | 0.23 | 4.2 (1.2) | 4.2 (1.2) | 0.01 |  |
| **HAS-BLED Score** including the index hospitalization and 3 years prior to the index claim | 3.2 (1.3) | 3.3 (1.3) | 0.08 | 3.2 (1.3) | 3.2 (1.3) | <0.01 |  |
| **Charlson Score Index (**including the index hospitalization and 3 years prior to the index claim) | 4.7 (3.5) | 4.8 (3.3) | 0.03 | 4.8 (3.4) | 4.7 (3.4) | 0.01 |  |
| **Frailty score** | 12.5 (6.8) | 13.6 (6.9) | 0.15 | 13.3 (6.8) | 13.2 (6.9) | <0.01 |  |
| **Comorbidities** (the index hospitalization and 3 years prior to the index claim)^¥^ - no (%) |  |  |  |  |  |  |  |
| Hypertension | 84.0 % | 83.8 % | 0.01 | 83.7% | 83.9% | <0.01 |  |
| Dyslipidemia | 50.5% | 49.0% | 0.03 | 49.3% | 49.6% | 0.01 |  |
| Diabetes | 31.1% | 28.8% | 0.05 | 30.0% | 29.7% | 0.01 |  |
| Coronary artery disease | 53.8% | 53.5% | 0.01 | 53.2% | 53.4% | <0.01 |  |
| Acute myocardial infarction | 14.4% | 16.5% | 0.06 | 15.8% | 15.8% | <0.01 |  |
| Chronic heart failure | 39.2% | 42.7% | 0.07 | 42.1% | 41.7% | 0.01 |  |
| Cardiomyopathy | 5.6% | 5.0% | 0.02 | 5.0% | 5.2% | 0.01 |  |
| Other cardiac rhythm disorders | 18.0% | 18.9% | 0.02 | 18.3% | 18.5% | 0.01 |  |
| Valvular disease | 19.4% | 21.9% | 0.06 | 21.1% | 21.2% | <0.01 |  |
| Prior cerebrovascular disease including TIA | 18.4% | 19.4% | 0.03 | 19.6% | 19.2% | 0.01 |  |
| Prior ischemic stroke | 17.8% | 18.6% | 0.02 | 18.9% | 18.5% | 0.01 |  |
| Peripheral vascular disease | 21.3% | 21.4% | <0.01 | 21.0% | 21.2% | <0.01 |  |
| Chronic renal failure | 40.4% | 45.3% | 0.10 | 43.3% | 43.5% | <0.01 |  |
| Chronic renal failure <30 mL/min | 3.0% | 3.6% | 0.03 | 3.7% | 3.4% | 0.01 |  |
| Acute renal failure | 24.9% | 28.1% | 0.07 | 27.0% | 27.1% | <0.01 |  |
| Chronic obstructive pulmonary disease/asthma | 37.7% | 34.3% | 0.07 | 35.0% | 35.2% | <0.01 |  |
| Liver disease | 1.9% | 1.5% | 0.03 | 1.5% | 1.7% | 0.01 |  |
| Systemic embolism | 2.1% | 1.9% | 0.01 | 1.9% | 1.9% | <0.01 |  |
| Depression | 12.6% | 12.3% | 0.01 | 12.3% | 12.4% | <0.01 |  |
| Hypothyroidism | 27.1% | 28.3% | 0.03 | 27.7% | 27.9% | <0.01 |  |
| Neurologic disorder | 27.3% | 31.1% | 0.08 | 29.4% | 29.9% | 0.01 |  |
| Prior major bleeding | 30.7% | 33.9% | 0.07 | 33.1% | 33.1% | <0.01 |  |
| Malignant cancer | 25.1% | 25.1% | <0.01 | 25.2% | 25.1% | <0.01 |  |
| **Medical procedures (in the 3 years prior to the index claim)** |  |  |  |  |  |  |  |
| Cardiac catheterization | 2.7% | 2.6% | 0.01 | 2.7% | 2.6% | <0.01 |  |
| Percutaneous coronary intervention - stent | 2.7% | 2.3% | 0.03 | 2.5% | 2.4% | 0.01 |  |
| Coronary artery bypass grafting | 0.6% | 0.2% | 0.06 | 0.3% | 0.3% | 0.01 |  |
| Implantable cardiac device | 0.1% | 0.0% | 0.03 | 0.02% | 0.00% | 0.02 |  |
| **Medications in the 2 weeks prior to the index claim** |  |  |  |  |  |  |  |
| Diuretics | 41.5% | 42.6% | 0.02 | 42.0% | 42.4% | 0.01 |  |
| Loop diuretics | 33.2% | 36.5% | 0.07 | 35.1% | 35.5% | 0.01 |  |
| B-blockers | 64.0% | 65.5% | 0.03 | 65.1% | 64.7% | 0.01 |  |
| Renin-angiotensin system Inhibitors | 37.1% | 35.2% | 0.04 | 35.3% | 35.6% | 0.01 |  |
| Calcium channel blockers | 38.4% | 39.5% | 0.02 | 38.9% | 39.2% | 0.01 |  |
| Statin | 42.0% | 41.3% | 0.01 | 40.8% | 41.3% | 0.01 |  |
| Antidiabetics | 17.4% | 16.7% | 0.02 | 17.2% | 17.0% | 0.01 |  |
| Antiplatelet agents (excluding low-dose ASA) | 5.4% | 5.9% | 0.02 | 5.7% | 5.7% | <0.01 |  |
| Low-dose ASA | 26.9% | 26.5% | 0.01 | 26.5% | 26.4% | <0.01 |  |
| Proton pump inhibitors | 42.4% | 44.0% | 0.03 | 43.7% | 43.6% | <0.01 |  |
| NSAIDs | 1.1% | 1.1% | 0.01 | 1.1% | 1.1% | <0.01 |  |
| Amiodarone | 9.6% | 10.1% | 0.02 | 9.6% | 9.8% | 0.01 |  |
| Digoxin | 11.6% | 10.2% | 0.04 | 11.1% | 10.9% | 0.01 |  |
| Antidepressant | 9.2% | 10.0% | 0.03 | 9.6% | 9.8% | <0.01 |  |
| PGP inhibitor use | 60.4% | 60.4% | <0.01 | 60.5% | 60.4% | <0.01 |  |
| Number of medications | 8.8 (4.2) | 9.1 (4.1) | 0.07 | 9.1 (4.1) | 9.0 (4.1) | 0.01 |  |
| **Medical service consumption in the year prior to the index claim** |  |  |  |  |  |  |  |
| Number of visits to a specialist, mean (SD) | 1.2 (2.1) | 1.3 (2.7) | 0.04 | 1.3 (2.9) | 1.3 (3.0) | 0.01 |  |
| Number of family physician visits, mean (SD) | 1.4 (3.0) | 1.3 (3.0) | 0.02 | 1.3 (2.5) | 1.3 (2.6) | <0.01 |  |
| Number of emergency room visits, mean (SD) | 3.3 (2.5) | 3.3 (2.6) | <0.01 | 3.3 (2.4) | 3.3 (2.6) | <0.01 |  |
| **Hospital service consumption in the 3 years prior to the index claim** |  |  |  |  |  |  |  |
| Mean number of all-cause hospital admission | 2.2 (1.7) | 2.2 (1.6) | 0.04 | 2.2 (1.6) | 2.2 (1.7) | <0.01 |  |
| Length of stay | 9.7 (11.0) | 10.5 (11.6) | 0.08 | 10.3 (11.5) | 10.3 (11.5) | 0.01 |  |

References used for the propensity score: 1) Mentias A et al., JAMA Network Open 2018; 1:e182870; 2) Lopes et al. Am J Med 2018; IPTW: inverse probability of treatment weighting.

**Supplemental Table S5.6.** The initial cohort vs. the cohort after IPTW, for the dabigatran 110 mg and rivaroxaban 15 mg groups.

|  | **Initial Cohort** | | | | **Cohort After IPTW** | | | |
| --- | --- | --- | --- | --- | --- | --- | --- | --- |
|  | Dabigatran  (n=1,929) | Rivaroxaban  (n=1,718) | Absolute standardized difference | Dabigatran  (n=1,929) | | Rivaroxaban  (n=1,718) | Absolute standardized difference |  |
| **Age** – mean (SD) | 81.2 (7.3) | 82.9 (7.2) | 0.23 | 81.9 (7.0) | | 82.0 (7.5) | 0.01 |  |
| **Female** sex | 57.9% | 61.3% | 0.07 | 59.6% | | 59.8% | <0.01 |  |
| **CHA2DS2-VAS Score** including the index hospitalization and 3 years prior to the index claim | 3.9 (1.3) | 4.0 (1.3) | 0.12 | 3.9 (1.2) | | 3.9 (1.3) | <0.01 |  |
| **HAS-BLED Score** including the index hospitalization and 3 years prior to the index claim | 3.0 (1.2) | 3.2 (1.3) | 0.12 | 3.0 (1.2) | | 3.1 (1.3) | 0.01 |  |
| **Charlson Score Index (**including the index hospitalization and 3 years prior to the index claim) | 4.2 (3.4) | 4.7 (3.5) | 0.13 | 4.4 (3.4) | | 4.4 (3.4) | 0.01 |  |
| **Frailty score** | 12.0 (6.8) | 12.5 (6.8) | 0.08 | 12.2 (6.8) | | 12.2 (6.7) | <0.01 |  |
| **Comorbidities** (the index hospitalization and 3 years prior to the index claim^¥^ - no (%) |  |  |  |  | |  |  |  |
| Hypertension | 82.6 % | 84.0 % | 0.04 | 83.2 % | | 82.9% | 0.01 |  |
| Dyslipidemia | 50.1% | 50.5% | 0.01 | 50.0% | | 49.7% | 0.01 |  |
| Diabetes | 30.6% | 31.1% | 0.01 | 30.5% | | 29.9% | 0.01 |  |
| Coronary artery disease | 51.8% | 53.8% | 0.04 | 52.5% | | 52.5% | <0.01 |  |
| Acute myocardial infarction | 9.6% | 14.4% | 0.15 | 11.9% | | 12.2% | 0.01 |  |
| Chronic heart failure | 35.3% | 39.2% | 0.08 | 36.7% | | 36.8% | <0.01 |  |
| Cardiomyopathy | 5.0% | 5.6% | 0.03 | 5.0% | | 4.9% | <0.01 |  |
| Other cardiac rhythm disorders | 23.2% | 18.0% | 0.13 | 20.7% | | 20.5% | <0.01 |  |
| Valvular disease | 16.8% | 19.4% | 0.07 | 18.2% | | 18.2% | <0.01 |  |
| Prior cerebrovascular disease including TIA | 22.9% | 18.4% | 0.11 | 20.7% | | 20.4% | 0.01 |  |
| Prior ischemic stroke | 22.6% | 17.8% | 0.12 | 20.3% | | 20.0% | 0.01 |  |
| Peripheral vascular disease | 18.7% | 21.3% | 0.06 | 20.2% | | 20.5% | 0.01 |  |
| Chronic renal failure | 25.3% | 40.4% | 0.33 | 32.7% | | 32.8% | <0.01 |  |
| Chronic renal failure <30 mL/min | 1.4% | 3.0% | 0.11 | 2.1% | | 2.2% | <0.01 |  |
| Acute renal failure | 12.8% | 24.9% | 0.31 | 18.6% | | 18.7% | <0.01 |  |
| Chronic obstructive pulmonary disease/asthma | 34.7% | 37.7% | 0.06 | 36.3% | | 36.1% | <0.01 |  |
| Liver disease | 1.9% | 1.9% | <0.01 | 2.0% | | 1.9% | <0.01 |  |
| Systemic embolism | 1.5% | 2.1% | 0.04 | 1.8% | | 1.8% | <0.01 |  |
| Depression | 13.6% | 12.6% | 0.03 | 13.1% | | 13.1% | <0.01 |  |
| Hypothyroidism | 22.1% | 27.1% | 0.12 | 24.6% | | 24.8% | 0.01 |  |
| Neurologic disorder | 26.4% | 27.3% | 0.03 | 27.1% | | 27.1% | <0.01 |  |
| Prior major bleeding | 27.7% | 30.7% | 0.07 | 29.3% | | 29.0% | 0.01 |  |
| Malignant cancer | 24.7% | 25.1% | 0.01 | 25.2% | | 25.1% | <0.01 |  |
| **Medical procedures (in the 3 years prior to the index claim)** |  |  |  |  | |  |  |  |
| Cardiac catheterization | 3.1% | 2.7% | 0.02 | 2.9% | | 2.8% | <0.01 |  |
| Percutaneous coronary intervention - stent | 2.1% | 2.7% | 0.04 | 2.4% | | 2.6% | 0.01 |  |
| Coronary artery bypass grafting | 0.6% | 0.6% | <0.01 | 0.6% | | 0.6% | <0.01 |  |
| Implantable cardiac device | 0.6% | 0.1% | 0.09 | 0.3% | | 0.3% | 0.01 |  |
| **Medications in the 2 weeks prior to the index claim** |  |  |  |  | |  |  |  |
| Diuretics | 36.7% | 41.5% | 0.10 | 38.8% | | 38.7% | <0.01 |  |
| Loop diuretics | 28.5% | 33.2% | 0.10 | 30.8% | | 30.8% | <0.01 |  |
| B-blockers | 62.2% | 64.0% | 0.04 | 62.9% | | 63.1% | <0.01 |  |
| Renin-angiotensin system Inhibitors | 41.1% | 37.1% | 0.08 | 39.2% | | 39.3% | <0.01 |  |
| Calcium channel blockers | 37.1% | 38.4% | 0.03 | 37.8% | | 37.5% | <0.01 |  |
| Statin | 43.8% | 42.0% | 0.03 | 42.8% | | 43.0% | 0.01 |  |
| Antidiabetics | 18.1% | 17.4% | 0.02 | 17.7% | | 17.1% | <0.01 |  |
| Antiplatelet agents (excluding low-dose ASA) | 4.8% | 5.4% | 0.03 | 5.1% | | 4.9% | 0.01 |  |
| Low-dose ASA | 27.9% | 26.9% | 0.02 | 27.7% | | 27.4% | 0.01 |  |
| Proton pump inhibitors | 42.8% | 42.4% | 0.01 | 43.1% | | 42.9% | <0.01 |  |
| NSAIDs | 1.6% | 1.1% | 0.05 | 1.3% | | 1.3% | <0.01 |  |
| Amiodarone | 10.0% | 9.6% | 0.01 | 10.1% | | 9.9% | 0.01 |  |
| Digoxin | 13.9% | 11.6% | 0.07 | 12.9% | | 12.9% | <0.01 |  |
| Antidepressant | 9.1% | 9.2% | <0.01 | 9.5% | | 9.2% | 0.01 |  |
| PGP inhibitor use | 58.0% | 60.4% | 0.05 | 59.5% | | 59.3% | <0.01 |  |
| Number of medications | 8.4 (4.1) | 8.8 (4.2) | 0.11 | 8.7 (4.2) | | 8.6 (4.2) | 0.01 |  |
| **Medical service consumption in the year prior to the index claim** |  |  |  |  | |  |  |  |
| Number of visits to a specialist, mean (SD) | 1.4 (3.0) | 1.2 (2.1) | 0.07 | 1.4 (3.1) | | 1.4 (3.0) | <0.01 |  |
| Number of family physician visits, mean (SD) | 1.5 (3.2) | 1.4 (3.0) | 0.03 | 1.3 (2.6) | | 1.3 (2.5) | 0.01 |  |
| Number of emergency room visits, mean (SD) | 3.2 (2.8) | 3.3 (2.5) | 0.03 | 3.2 (2.8) | | 3.2 (2.5) | <0.01 |  |
| **Hospital service consumption in the 3 years prior to the index claim** |  |  |  |  | |  |  |  |
| Mean number of all-cause hospital admission | 2.3 (1.7) | 2.2 (1.7) | 0.04 | 2.3 (1.6) | | 2.3 (1.7) | <0.01 |  |
| Length of stay | 9.1 (11.0) | 9.7 (11.0) | 0.05 | 9.4 (11.0) | | 9.3 (10.9) | 0.01 |  |

References used for the propensity score: 1) Mentias A et al., JAMA Network Open 2018; 1:e182870; 2) Lopes et al. Am J Med 2018; IPTW: inverse probability of treatment weighting.
