# Supplementary material for: Comparative Effectiveness and Safety of Low-Dose Oral Anticoagulants in Patients With Atrial Fibrillation
Source: Front Pharmacol. 2022 Jan 14;12:812018. doi: 10.3389/fphar.2021.812018 (PMC8795908; doi:10.3389/fphar.2021.812018)
Supplement: Supplementary file 7 [file Table3.docx]

**Supplementary Tables:**

**Supplemental Table S3.** Definitions of the variables used in the CHA_2_DS_2_-VASc risk score, according to the ICD-9 and ICD-10 codes recorded in the Med-Echo databases.

|  | ICD-9 codes | ICD-10 codes |
| --- | --- | --- |
| **CHA_2_DS_2_-VASc** |  |  |
| Congestive heart failure | 402.01, 402.11, 402.91, 404.01, 404.11, 404.91, 404.03, 404.13, 404.93, 425.4,428.0 | I11.0, I13.0, I13.2, I42.0, I50 |
| Left ventricular dysfunction | 428.1, 428.9 | I50.1, I50.9 |
| Hypertension | 401 | I10 |
| Diabetes | 250.x | E08, E10, E11, E13 |
| Ischemic stroke | 433.xx, 434.xx, 436 | I63 except 63.6, I67.89 |
| Systemic embolism | 444.x, 557.0, 362.31, 362.32, 598.31 | I74, K55.0, H34.1, H34.2, N28.0 |
| Transient ischemic accident | 435.x | G45 |
| Aortic plaque | 440.0 | I70.0 |
| Peripheral arterial disease | 440 (except 440.0), 441, 443.0, 443.89, 443.9 | I70.1 to I70.9, I71, I73.0, I73.89, I73.9 |
| Myocardial infarction | 410.xx | I21, I22, I23 |
