# Supplementary material for: Comparative Effectiveness and Safety of Low-Dose Oral Anticoagulants in Patients With Atrial Fibrillation
Source: Front Pharmacol. 2022 Jan 14;12:812018. doi: 10.3389/fphar.2021.812018 (PMC8795908; doi:10.3389/fphar.2021.812018)
Supplement: Supplementary file 8 [file Table4.docx]

**Supplementary Tables:**

**Supplemental Table S4.** Definition of variables used in the HAS-BLED risk score, based on associated morbidities and concomitant drugs.

|  | **ICD-9** | **ICD-10** |
| --- | --- | --- |
| **Modified HAS-BLED** |  |  |
| Hypertension | 401 | I10 |
| Moderate-to-severe renal disease | 404.01, 404.03, 404.11, 404.13, 404.91, 404.93, 580.0, 580.4, 581.0, 581.1, 581.2, 581.3, 581.89, 581.9, 582.0, 582.1, 582.2, 582.89, 582.9, 583.0, 583.1, 583.2, 583.4, 583.7, 583.6, 583.89, 583.9, 584.5, 584.6, 584.7, 584.8, 584.9, 585.1, 585.2, 585.3, 585.4, 585.5, 585.6, 586, 590.0, 590.01, 590.80 | I12, I13, N00, N01, N02, N03, N04, N05, N07, N11, N12, N14, N17, N18, N19 |
| Moderate-to-severe liver disease | 570, 572.3, 070.0, 070.21, 070.20, 070.60 | K7200, K762, K766, B150, B160, B162, B190, K704, I85 |
| Intracranial hemorrhagic stroke (non-traumatic) | 430, 431, 432.x | I60 , I61, I62 |
| Extracranial major bleeding or unclassified major bleeding | Upper GI: 456.1, 530.7, 531.0x, 531.2x, 531.4x, 531.6x, 532.0x, 532.2x, 532.4x. 532.6x, 533.0x. 533.2x, 533.4x, 533.6x, 534.0x, 534.2x, 534.4x, 534.6x, 535.01, 537.83, 578.0  Lower GI: 562.02, 562.03, 562.12, 562.13, 569.3x, 569.85, 578.1x, 578.9  Other sites:  626.2x and 280.0, 285.1 or 285.9  599.7, 786.3x, 379.23, 719.1x, 423.0x, 568.8, 459.0x, 285.1x | Upper GI: I850, K226, K250, K252, K254, K256, K260, K262, K264, K266, K270, K272, K274, K276, K280, K282, K284, K286, K2901, K290, K31811, K920  Lower GI: K921, K922, K5711, K5713, K5731, K5733, K625, K5521  Other sites:  N92.0 and D50.0 or D62 or D64.9,  R31, R042, R0489, R049, H43.13,  M250x, I31.2, K66.1, R58.0, D62 |
| Gastrointestinal bleeding | Upper GI: 531.0x, 531.2x, 531.4x, 531.6x, 532.0x, 532.2x, 532.4x. 532.6x, 533.0x. 533.2x, 533.4x, 533.6x, 534.0x, 534.2x, 534.4x, 534.6x, 535.01, 537.83, 578.0  Lower GI: 562.02, 562.03, 562.12, 562.13, 569.3x, 569.85, 578.1x, 578.9 | Upper GI: K250, K252, K254, K256, K260, K262, K264, K266, K270, K272, K274, K276, K280, K282, K284, K286, K2901, K290, K31811, K920  Lower GI: K921, K922, K5711, K5713, K5731, K5733, K625, K5521 |
| Traumatic intracranial bleeding | 852x, 853x | S063, S064, S065, S066 |
| Clopidogrel, ticlopidine, prasugrel, ticagrelor | 46486, 47307, 45617, 47402, 47834, 47866 | 46486, 47307, 45617, 47402, 47834, 47866 |
| Low-dose ASA | 00143, 46353 (daily dose < 100 mg) | 00143, 46353 (daily dose < 100 mg) |
| Nonsteroidal anti-inflammatory drugs | 46353, 38184, 47327, 47078, 41694, 47059, 43150, 47122, 33803, 44749, 04745, 46654, 47506, 04810, 38691, 44359, 47385, 47084, 19752, 47890, 07462, 42019, 47346, 47107, 40381, 45592, 45407, 03766 | 46353, 38184, 47327, 47078, 41694, 47059, 43150, 47122, 33803, 44749, 04745, 46654, 47506, 04810, 38691, 44359, 47385, 47084, 19752, 47890, 07462, 42019, 47346, 47107, 40381, 45592, 45407, 03766 |
| Alcohol | 331.7, 359.4, 425.5, 577.1 | E224, E529A, F10, G312, G612, G721, I426, K292, K70, K860, L278A, O354, T51, Z714, Z721 |
