# Supplementary material for: Comparative Effectiveness and Safety of Low-Dose Oral Anticoagulants in Patients With Atrial Fibrillation
Source: Front Pharmacol. 2022 Jan 14;12:812018. doi: 10.3389/fphar.2021.812018 (PMC8795908; doi:10.3389/fphar.2021.812018)
Supplement: Supplementary file 9 [file Table6.docx]

**Supplementary Tables:**

**Supplemental Table S6.1.** Rates of incident outcomes for each low-dose DOAC versus warfarin in an as-treated analysis after IPTW.

|  |  |  |  |  |  |  |  |
| --- | --- | --- | --- | --- | --- | --- | --- |
|  | | **IPTW dabigatran and warfarin populations** | | **IPW rivaroxaban and warfarin populations** | | **IPTW apixaban and warfarin populations** | |
|  | | Dabigatran |  | Rivaroxaban |  | Apixaban |  |
|  |  | 110 mg twice daily | Warfarin | 15 mg once daily | Warfarin | 2.5 mg twice daily | Warfarin |
|  |  | (n=1,926) | (n=14,700) | (n=1,717) | (n=14,700) | (n=3,815) | (n=14,700) |
| **Effectiveness** | |  |  |  |  |  |  |
| Stroke/SE | |  |  |  |  |  |  |
| Events | | 16.7 | 148.8 | 19.8 | 149.7 | 51.2 | 153.5 |
| Event rate per 100 PY^‡^ | | 1.30 (0.67-1.92) | 1.53 (1.28-1.78) | 1.67 (0.93-2.41) | 1.54 (1.29-1.79) | 1.94 (1.40-2.46) | 1.59 (1.34-1.84) |
| Hazard Ratio (95%CI)* | | 0.85 (0.51-1.40) | | 1.10 (0.69-1.75) | | 1.24 (0.91-1.71) | |
| Death | |  |  |  |  |  |  |
| Events | | 23.1 | 378.2 | 30.6 | 386.5 | 94.7 | 409.0 |
| Event rate per 100 PY ^‡^ | | 1.78 (1.06-2.52) | 3.87 (3.48-4.26) | 2.57(1.66-3.48) | 3.97 (3.57-4.36) | 3.56 (2.84-4.28) | 4.22 (3.81-4.63) |
| Hazard Ratio (95%CI)* | | 0.46 (0.30-0.70) | | 0.65 (0.45-0.94) | | 0.85 (0.68-1.06) | |
| Effectiveness composite | |  |  |  |  |  |  |
| Events | | 39.8 | 512.4 | 49.6 | 521.8 | 141.7 | 546.6 |
| Event rate per 100 PY^‡^ | | 3.09 (2.13-4.05) | 5.27 (4.81-5.72) | 4.18 (3.02-5.35) | 5.37 (4.91-5.83) | 5.35 (4.47-6.24) | 5.66 (5.18-6.13) |
| Hazard Ratio (95%CI)* | | 0.59 (0.42-0.81) | | 0.78 (0.59-1.05) | | 0.96 (0.80-1.15) | |
| **Safety** | |  | | | | | |
| Safety composite | |  |  |  |  |  |  |
| Events | | 51.8 | 362.4 | 48.1 | 363.5 | 68.6 | 369.4 |
| Event rate per 100 PY^‡^ | | 4.01 (2.93-5.12) | 3.74 (3.35-4.12) | 4.09 (2.93-5.25) | 3.75 (3.37-4.14) | 2.59 (1.98-3.20) | 3.83 (3.44-4.23) |
| Hazard Ratio (95%CI)* | | 1.07 (0.80-1.44) | | 1.10 (0.81-1.48) | | 0.68 (0.53-0.88) | |
| Intracranial bleeding | |  |  |  |  |  |  |
| Events | | 9.8 | 107.2 | 5.3 | 106.9 | 20.9 | 111.3 |
| Event rate per 100 PY^‡^ | | 0.76 (0.28-1.23) | 1.10 (0.89-1.31) | 0.44 (0.07-0.82) | 1.10 (0.89-1.31) | 0.79 (0.45-1.12) | 1.15 (0.93-1.36) |
| Hazard Ratio (95%CI)* | | 0.69 (0.36-1.32) | | 0.41 (0.17-0.97) | | 0.69 (0.43-1.09) | |
| Gastrointestinal bleeding | |  |  |  |  |  |  |
| Events | | 24.1 | 139.2 | 23.5 | 139.6 | 29.0 | 140.2 |
| Event rate per 100 PY^‡^ | | 1.87 (1.12-2.62) | 1.43 (1.19-1.67) | 1.98 (1.18-2.79) | 1.44 (1.20-1.67) | 1.09 (0.69-1.49) | 1.45(1.21-1.69) |
| Hazard Ratio (95%CI)* | | 1.31 (0.85-2.01) | | 1.40 (0.90-2.16) | | 0.77 (0.52-1.15) | |
| Composite benefit/risk^†^ | |  |  |  |  |  |  |
| Events | | 90.5 | 854.3 | 96.7 | 864.5 | 204.8 | 894.9 |
| Event rate per 100 PY^‡^ | | 7.06 (6.58-9.70) | 8.84 (9.98-11.28) | 8.25 (9.08-12.89) | 8.96 (10.15-11.46) | 7.77 (8.42-10.79) | 9.32 (10.56-11.90) |
| Hazard Ratio (95%CI)* | | 0.80 (0.64-0.99) | | 0.93 (0.75-1.14) | | 0.84 (0.73-0.98) | |

^‡^ :PY: Person-Years; ***** CI: confidence interval; † benefit/risk composite: stroke/SE, all-cause mortality, and major bleeding.

**Supplemental Table S6.2.** Rates of incident outcomes for each low-dose DOAC versus warfarin as intention-to-treat (ITT) after IPTW.

|  | **IPTW Dabigatran and warfarin population** | | **IPW Rivaroxaban and warfarin population** | | **IPTW Apixaban and warfarin population** | |
| --- | --- | --- | --- | --- | --- | --- |
|  | Dabigatran |  | Rivaroxaban |  | Apixaban |  |
|  | 110 mg twice daily | Warfarin | 15 mg once daily | Warfarin | 2.5 mg twice daily | Warfarin |
|  | (n=1,926) | (n=14,700) | (n=1,717) | (n=14,700) | (n=3,815) | (n=14,700) |
| **Effectiveness** |  |  |  |  |  |  |
| Stroke/SE |  |  |  |  |  |  |
| Events | 19.5 | 188.7 | 26.7 | 189.9 | 56.3 | 193.6 |
| Event rate per 100 PY^‡^ | 1.20 (0.67-1.74) | 1.58 (1.36-1.81) | 1.95 (1.21-2.69) | 1.60 (1.37-1.82) | 1.89 (1.40-2.39) | 1.64 (1.41-1.87) |
| Hazard Ratio (95%CI)* | 0.77 (0.48-1.22) | | 1.22 (0.81-1.83) | | 1.15 (0.86-1.55) | |
| Death |  |  |  |  |  |  |
| Events | 138.8 | 1,307.7 | 156.8 | 1341.1 | 395.0 | 1,425.3 |
| Event rate per 100 PY^‡^ | 8.55 (7.13-9.97) | 10.91 (10.32-11.51) | 11.41 (9.62-13.19) | 11.23 (10.63-11.83) | 13.22 (11.91-14.52) | 12.03 (11.41-12.66) |
| Hazard Ratio (95%CI)* | 0.79 (0.66-0.94) | | 1.01 (0.86-1.20) | | 1.10 (0.98-1.23) | |
| Effectiveness composite |  |  |  |  |  |  |
| Events | 156.7 | 1,456.4 | 178.1 | 1,490.1 | 441.6 | 1,573.7 |
| Event rate per 100 PY^‡^ | 9.68 (8.16-11.19) | 12.21 (11.58-12.84) | 13.00 (11.10-14.92) | 12.54 (11.90-13.17) | 14.86 (13.47-16.25) | 13.34 (12.69-14.00) |
| Hazard Ratio (95%CI)* | 0.80 (0.68-0.94) | | 1.04 (0.89-1.21) | | 1.11 (1.00-1.24) | |
| **Safety** |  | | | | | |
| Safety composite |  |  |  |  |  |  |
| Events | 64.4 | 454.0 | 59.0 | 455.5 | 84.1 | 460.4 |
| Event rate per 100 PY^‡^ | 4.01 (3.05-5.00) | 3.84 (3.48-4.19) | 4.37 (3.26-5.49) | 3.86 (3.51-4.22) | 2.84 (2.23-3.44) | 3.94 (3.58-4.30) |
| Hazard Ratio (95%CI)* | 1.05 (0.81-1.36) | | 1.13 (0.86-1.48) | | 0.72 (0.57-0.91) | |
| Intracranial bleeding |  |  |  |  |  |  |
| Events | 10.1 | 125.9 | 7.0 | 125.4 | 26.2 | 130.4 |
| Event rate per 100 PY^‡^ | 0.62 (0.24-1.01) | 1.05 (0.87-1.24) | 0.51 (0.13-0.89) | 1.05 (0.87-1.24) | 0.88 (0.54-1.21) | 1.10 (0.91-1.29) |
| Hazard Ratio (95%CI)* | 0.59 (0.31-1.12) | | 0.49 (0.23-1.04) | | 0.89 (0.59-1.32) | |
| Gastrointestinal bleeding |  |  |  |  |  |  |
| Events | 26.6 | 188.3 | 26.7 | 188.9 | 32.5 | 187.0 |
| Event rate per 100 PY^‡^ | 1.65 (1.02-2.27) | 1.58 (1.35-1.81) | 1.96 (1.22-2.71) | 1.59 (1.36-1.82) | 1.09 (0.72-1.47) | 1.59 (1.36-1.82) |
| Hazard Ratio (95%CI)* | 1.05 (0.70-1.58) | | 1.23 (0.82-1.84) | | 0.69 (0.48-0.99) | |
| Composite benefit/risk^†^ |  |  |  |  |  |  |
| Events | 211.4 | 1,828.0 | 231.1 | 1,862.9 | 492.6 | 1,946.7 |
| Event rate per 100 PY^‡^ | 13.23 (12.44-16.16) | 15.52 (16.29-17.79) | 17.20 (17.16-21.91) | 15.87 (16.68-18.20) | 16.71 (16.62-19.70) | 16.72 (17.57-19.13) |
| Hazard Ratio (95%CI)* | 0.86 (0.74-0.99) | | 1.08 (0.94-1.24) | | 1.00 (0.91-1.10) | |

^‡^ :PY: Person-Years; ***** CI: confidence interval; † benefit/risk composite: stroke/SE, all-cause mortality, and major bleeding.

**Supplemental Table S6.3.** Rates of incident outcomes for each low-dose DOAC versus other DOACs in an as-treated analysis, after IPTW.

|  | **IPTW Dabigatran and**  **Apixaban populations** | | **IPTW Rivaroxaban and Apixaban populations** | | **IPTW Dabigatran and Rivaroxaban populations** | |
| --- | --- | --- | --- | --- | --- | --- |
|  | Dabigatran | Apixaban | Rivaroxaban | Apixaban | Dabigatran | Rivaroxaban |
|  | 110 mg twice daily | 2.5 mg twice daily | 15 mg once daily | 2.5 mg twice daily | 110 mg twice daily | 15 mg once daily |
|  | (n=1,926) | n=(3,815) | (n=1,717) | n=(3,815) | (n=1,926) | (n=1,717) |
| **Effectiveness** |  | | | | | |
| Stroke/SE |  |  |  |  |  |  |
| Events | 15.5 | 63.5 | 18.2 | 60.5 | 15.2 | 17.5 |
| Event rate per 100 PY^‡^ | 1.25 (0.63-1.87) | 2.28 (1.72-2.83) | 1.55 (0.84-2.27) | 2.21 (1.66-2.77) | 1.17 (0.58-1.76) | 1.45 (0.77-2.13) |
| Hazard Ratio (95%CI)* | 0.53 (0.30-0.93) | | 0.70 (0.41-1.17) | | 0.80 (0.40-1.59) | |
| Death |  |  |  |  |  |  |
| Events | 18.6 | 94.0 | 40.8 | 100.8 | 23.0 | 30.8 |
| Event rate per 100 PY^‡^ | 1.50 (0.82-2.18) | 3.35 (2.67-4.02) | 3.48 (2.41-4.55) | 3.67 (2.95-4.38) | 1.77 (1.05-2.49) | 2.54 (1.65-3.44) |
| Hazard Ratio (95%CI)* | 0.43 (0.26-0.71) | | 0.94 (0.65-1.35) | | 0.68 (0.40-1.17) | |
| Effectiveness composite |  |  |  |  |  |  |
| Events | 34.1 | 151.7 | 57.8 | 155.0 | 38.2 | 47.1 |
| Event rate per 100 PY^‡^ | 2.75 (1.83-3.67) | 5.43 (4.57-6.30) | 4.94 (3.67-6.21) | 5.67 (4.78-6.56) | 2.95 (2.01-3.88) | 3.90 (2.79-5.02) |
| Hazard Ratio (95%CI)* | 0.49 (0.34-0.71) | | 0.86 (0.64-1.17) | | 0.75 (0.49-1.14) | |
| **Safety** |  | | | | | |
| Safety composite |  |  |  |  |  |  |
| Events | 60.0 | 66.6 | 46.7 | 69.1 | 56.3 | 44.3 |
| Event rate per 100 PY^‡^ | 4.85 (3.62-6.08) | 2.38 (1.81-2.95) | 4.02 (2.87-5.17) | 2.53 (1.93-3.12) | 4.35 (3.22-5.49) | 3.70 (2.61-4.79) |
| Hazard Ratio (95%CI)* | 2.02 (1.42-2.86) | | 1.58 (1.09-2.29) | | 1.16 (0.79-1.72) | |
| Intracranial bleeding |  |  |  |  |  |  |
| Events | 11.9 | 19.8 | 12.7 | 19.8 | 10.4 | 5.7 |
| Event rate per 100 PY^‡^ | 0.96 (0.41-1.50) | 0.71 (0.39-1.02) | 1.09 (0.49-1.68) | 0.72 (0.40-1.04) | 0.80 (0.31-1.29) | 0.47 (0.08-0.86) |
| Hazard Ratio (95%CI)* | 1.35 (0.65-2.76) | | 1.51 (0.75-3.05) | | 1.70 (0.61-4.74) | |
| Gastrointestinal bleeding |  |  |  |  |  |  |
| Events | 30.0 | 27.1 | 16.9 | 27.6 | 27.8 | 20.3 |
| Event rate per 100 PY^‡^ | 2.42 (1.55-3.29) | 0.97 (0.60-1.33) | 1.44 (0.76-2.14) | 1.01 (0.63-1.38) | 2.15 (1.35-2.95) | 1.69 (0.95-2.42) |
| Hazard Ratio (95%CI)* | 2.47 (1.47-4.16) | | 1.42 (0.77-2.60) | | 1.26 (0.71-2.24) | |
| Composite benefit/risk^†^ |  |  |  |  |  |  |
| Events | 93.3 | 215.0 | 101.0 | 220.9 | 93.5 | 89.5 |
| Event rate per 100 PY^‡^ | 7.56 (7.03-10.33) | 7.74 (8.19-10.46) | 8.71 (8.68-12.43) | 8.11 (8.61-10.97) | 7.26 (6.82-9.99) | 7.50 (7.84-11.37) |
| Hazard Ratio (95%CI)* | 0.96 (0.75-1.22) | | 1.06 (0.84-1.35) | | 0.96 (0.72-1.28) | |

^‡^ :PY: Person-Years; ***** CI: confidence interval; † benefit/risk composite: stroke/SE, all-cause mortality, and major bleeding.

**Supplemental Table S6.4.** Rates of incident outcomes for each low-dose DOAC versus other DOACs in an intention-to-treat analysis after IPTW.

|  | **IPTW dabigatran and**  **Apixaban populations** | | **IPTW rivaroxaban and apixaban populations** | | **IPTW dabigatran and rivaroxaban populations** | |
| --- | --- | --- | --- | --- | --- | --- |
|  | Dabigatran | Apixaban | Rivaroxaban | Apixaban | Dabigatran | Rivaroxaban |
|  | 110 mg twice daily | 2.5 mg twice daily | 15 mg once daily | 2.5 mg twice daily | 110 mg twice daily | 15 mg once daily |
|  | (n=1,926) | n=(3,815) | (n=1,717) | n=(3,815) | (n=1,926) | (n=1,717) |
| **Effectiveness** |  | | | | | |
| Stroke/SE |  |  |  |  |  |  |
| Events | 19.3 | 71.2 | 25.9 | 68.8 | 20.0 | 25.3 |
| Event rate per 100 PY^‡^ | 1.25 (0.69-1.81) | 2.27 (1.74-2.80) | 1.92 (1.18-2.65) | 2.24 (1.71-2.77) | 1.24 (0.69-1.78) | 1.78 (1.09-2.48) |
| Hazard Ratio (95%CI)* | 0.55 (0.34-0.92) | | 0.85 (0.54-1.34) | | 0.70 (0.39-1.25) | |
| Death |  |  |  |  |  |  |
| Events | 138.2 | 394.1 | 184.0 | 411.5 | 132.2 | 136.0 |
| Event rate per 100 PY^‡^ | 8.94 (7.45-10.43) | 12.48 (11.25-13.71 | 13.57 (11.61-15.53) | 13.31 (12.02-14.60) | 8.16 (6.76-9.54) | 9.55 (7.94-11.15) |
| Hazard Ratio (95%CI)* | 0.72 (0.59-0.87) | | 1.02 (0.85-1.21) | | 0.86 (0.67-1.09) | |
| Effectiveness composite |  |  |  |  |  |  |
| Events | 156.5 | 453.3 | 206.1 | 467.0 | 150.6 | 157.9 |
| Event rate per 100 PY^‡^ | 10.15 (8.56-11.74) | 14.45 (13.12-15.78) | 15.24 (13.16-17.32) | 15.19 (13.82-16.57) | 9.32 (7.83-10.80) | 11.12 (9.39-12.86) |
| Hazard Ratio (95%CI)* | 0.70 (0.59-0.84) | | 1.00 (0.85-1.18) | | 0.84 (0.67-1.05) | |
| **Safety** |  | | | | | |
| Safety composite |  |  |  |  |  |  |
| Events | 67.0 | 77.0 | 55.9 | 79.5 | 65.5 | 53.6 |
| Event rate per 100 PY^‡^ | 4.39 (3.34-5.44) | 2.45 (1.91-3.00) | 4.19 (3.09-5.29) | 2.59 (2.02-3.16) | 4.10 (3.10-5.09) | 3.83 (2.80-4.85) |
| Hazard Ratio (95%CI)* | 1.79 (1.29-2.49) | | 1.61 (1.14-2.27) | | 1.07 (0.75-1.54) | |
| Intracranial bleeding |  |  |  |  |  |  |
| Events | 12.4 | 23.7 | 14.2 | 23.8 | 11.2 | 7.9 |
| Event rate per 100 PY^‡^ | 0.80 (0.36-1.25) | 0.75 (0.45-1.05) | 1.05 (0.50-1.59) | 0.77 (0.46-1.08) | 0.69 (0.29-1.10) | 0.56 (0.17-0.94) |
| Hazard Ratio (95%CI)* | 1.07 (0.54-2.12) | | 1.36 (0.70-2.62) | | 1.24 (0.50-3.08) | |
| Gastrointestinal bleeding |  |  |  |  |  |  |
| Events | 33.5 | 32.3 | 23.5 | 33.3 | 31.5 | 25.4 |
| Event rate per 100 PY^‡^ | 2.18 (1.44-2.92) | 1.03 (0.67-1.38) | 1.75 (1.04-2.46) | 1.08 (0.71-1.45) | 1.96 (1.27-2.64) | 1.80 (1.10-2.50) |
| Hazard Ratio (95%CI)* | 2.12 (1.31-3.45) | | 1.61 (0.95-2.73) | | 1.09 (0.65-1.84) | |
| Composite benefit/risk^†^ |  |  |  |  |  |  |
| Events | 210.1 | 507.2 | 250.2 | 523.7 | 205.5 | 204.3 |
| Event rate per 100 PY^‡^ | 13.81 (12.84-16.71) | 16.28 (16.05-19.00) | 18.82 (18.00-22.88) | 17.17 (16.97-20.03) | 12.89 (12.07-15.74) | 14.64 (14.29-18.56) |
| Hazard Ratio (95%CI)* | 0.85 (0.72-0.99) | | 1.09 (0.94-1.27) | | 0.88 (0.73-1.07) | |

^‡^ :PY: Person-Years; ***** CI: confidence interval; † benefit/risk composite: stroke/SE, all-cause mortality, and major bleeding.
